# Refits, cobbles, and fire: Approaching the temporal nature of an expedient Gravettian lithic assemblage from Lagar Velho (Leiria, Portugal)

**DOI:** 10.1371/journal.pone.0294866

**Published:** 2023-12-20

**Authors:** Elvira Susana Alonso-Fernández, Manuel Vaquero, Joan Daura, Ana Maria Costa, Montserrat Sanz, Ana Cristina Araújo

**Affiliations:** 1 Institut Català de Paleoecologia Humana i Evolució Social (IPHES), Tarragona, Spain; 2 Àrea de Prehistòria, Universitat Rovira i Virgili (URV), Tarragona, Spain; 3 Department of History and Archaeology, Grup de Recerca del Quaternari, GRQ‑SERP, Universitat de Barcelona, Barcelona, Spain; 4 Faculdade de Letras, UNIARQ‑Centro de Arqueologia da Universidade de Lisboa, Universidade de Lisboa, Lisbon, Portugal; 5 Laboratório de Arqueociências (LARC)-DGPC, Lisbon, Portugal; 6 InBIO Laboratório Associado, BIOPOLIS ‑- Programme in Genomics, Biodiversity and Land Planning, CIBIO—Centro de Investigação em Biodiversidade e Recursos Genéticos, Vairão, Portugal; 7 IDL ‑- Instituto Dom Luiz, Universidade de Lisboa, Lisbon, Portugal; 8 IIIPC ‑- Instituto Internacional de Investigaciones Prehistóricas de Cantabria, Universidad de Cantabria—Gobierno de Cantabria, Santander, Spain; Universita degli Studi di Ferrara, ITALY

## Abstract

Upper Paleolithic lithic assemblages have traditionally been considered a paramount example of the high level of complexity characterizing the technological behavior of prehistoric modern humans. The diversity and standardization of tools, as well as the systematic production of blades and bladelets, show the high investment of time, energy and knowledge often associated with Upper Paleolithic technocomplexes. However, more expedient behaviors have also been documented. In some cases, such low-cost behaviors can be dominant or almost exclusive, giving assemblages of Upper Paleolithic age an “archaic” appearance. In this paper, we address these expedient Upper Paleolithic technologies through the study of a lithic assemblage recovered from a Gravettian-age layer from the Lagar Velho rockshelter (Leiria, Portugal). Due to the specific formation processes characterizing this site, we also discuss the distinction between artifacts and geofacts, an aspect that is particularly difficult in expedient assemblages. Moreover, the combination of lithic refitting and data on thermal damage allows us to approach the temporal nature of the lithic assemblage and the timing of the different agents contributing to its formation.

## Introduction

The complexity of technical behaviors is one of the main factors underpinning the variability of prehistoric lithic assemblages. Although different concepts of complexity can be proposed [1–4], an operational definition must consider the amount of time, energy and knowledge invested in lithic production. This technological investment is expressed in all the stages of the production sequence, from raw material provisioning to artifact use and abandonment, as well as in the learning and knowledge transmission processes. Traditional evolutionist perspectives argued that prehistoric technological evolution could be characterized as a sequence of increasing complexity, from the simple behaviors evident in the first anthropogenic lithic assemblages to the complex technological systems of the most recent hunter-gatherer societies. This idea of cumulative culture was more or less explicitly related to human biological evolution and the consequent increase in cognitive capabilities. Although this unilinear view can still be considered valid in a coarse-grained approach, the study of lithic assemblages indicates that the reality is not this straightforward, and several apparent steps back have been identified. This highlights the fact that the degree of technological complexity not only depends on human cognitive abilities, but also on a wide range of factors related to the economic, social, cultural, and demographic realms.

One of the best examples of this is the persistence of expedient, low-cost technologies in the Pleistocene and Holocene. Technologies characterized by low investments of time and effort in terms of lithic production have been well documented from Lower and Middle Paleolithic assemblages generated by ‘archaic humans’, but also in Upper Paleolithic and Mesolithic technocomplexes associated with ‘modern humans’. This is particularly significant in the case of the Upper Paleolithic, when the highest levels of cultural and technological complexity in hunter-gatherer prehistory were achieved. In lithic technology, such complexity is evident from the development of blade and bladelet production strategies and the manufacture of a wide array of specialized and standardized tools. In this context, the assemblages indicating the use of expedient technologies in Upper Paleolithic times deserve special attention. In general, this expedient behavior can be seen in all actions related to lithic production and it has three main characteristics: 1) the use of strictly local raw materials, regardless of their suitability for knapping; 2) the production of flakes through reduction strategies where neither predetermination nor core preparation is evident; 3) the manufacture of simple and non-standardized toolkits, in which notches and denticulates are often predominant within the group of retouched pieces [5–9].

In Upper Paleolithic assemblages, these low-cost technologies have been recognized in a number of contexts [10–15]. Most commonly, an expedient component coexists with artifacts indicating more elaborate strategies aimed at producing blades and/or bladelets. These expedient assemblages present a wide variability in reduction strategies (unidirectional, orthogonal, multidirectional, centripetal) where the common feature is the systematic adaptation to the size and morphology of the knapped nodules, without any evidence indicating the search for a standardized or predetermined core structure. In some instances, this expedient component shows a considerable temporal persistence. In Portugal, expedient flake-production has been recorded during the Upper Paleolithic and related to non-flint raw materials using unidirectional and centripetal reduction strategies [16,17]. The exploitation of these local coarse-grained rocks was normally aimed at the production of flakes, mostly used unretouched. This pattern is seen particularly in the sites of the Côa Valley, but also in southern and central Portugal [18]. For example, the importance of simple and expedient debitage production has been highlighted in both the Gravettian and Solutrean layers from Vale Boi [19,20] and broadly identified from other Portuguese sites in the region of the Douro River [21–24]. As these low-cost behaviors increase and become predominant, or even exclusive, the chrono-cultural adscription of the lithic assemblages is more difficult and even their Upper Paleolithic character becomes blurred.

In a previous paper [25], we suggested that this increase of expedient technologies would be more likely in periods of cultural change, in which the old technological rules were vanishing, but the new ones were not yet fully established. This would be consistent with higher expediency indices during the chrono-cultural boundaries between the main stages of the Upper Paleolithic. This is the case of the Solutrean-Magdalenian boundary with the appearance of the Badegoulian technocomplex, characterized by the common use of expedient flake production strategies and a marked decrease in the manufacturing of blades and bladelets [13,26–28]. On the Iberian Peninsula, the end of the Magdalenian traditions is defined by the sudden emergence of expedient technocomplexes–Mirean, Languedocean, Ancorean, Asturian, Mesolithic of denticulates and notches–at the beginning of the Holocene. These assemblages show all the typical features of low-cost technologies and a nearly complete absence of the toolkits and laminar production methods that defined the end of the Upper Paleolithic [29–35]. In addition, this decrease in technological complexity was also evident in other domains of the material culture, such as the bone industry and symbolic expression, indicating that low levels of technical investment were not exclusive to lithic production, but actually permeated the entire technological system.

This rise of expedient behaviors was not as clear-cut during the Gravettian-Solutrean boundary, although we must bear in mind the fact that several assemblages apparently corresponding to this moment present serious problems on terms of their chronology and/or technological characterization. However, expedient trends have been observed at some sites in the Cantabrian region dated to this time interval. In north-western Iberia, layer D in Valdavara cave yielded a lithic assemblage showing a marked dominance of expedient reduction strategies applied to local quartz [25]. Flake cores are predominant and denticulates relatively abundant in layer I of La Riera cave, also characterized by the prevailing use of local quartzite [36]. In comparison with the underlying units, the use of quartz and the proportion of flake cores increase in layer III of Aitzbitarte III [37]. An expedient component related to quartzite coexisting with a flint blade and bladelet production has also been identified at layer V of Llonín Cave [38]. However, the best example of a low-cost assemblage from the Gravettian-Solutrean boundary was found at Esquilleu cave. The top of the sequence–especially layer III–exhibits a marked decrease in technological complexity compared to the underlying units. Moreover, laminar production and Upper Paleolithic tools are totally absent [39,40]. Although these assemblages have been considered to be Middle Paleolithic, there is a possibility that they are, in fact, Upper Paleolithic [41].

A variety of causes can be invoked to explain this apparent coexistence or contemporaneity of expedient and more elaborate technologies in Upper Paleolithic times. Firstly, the differential investment in lithic production could be related to diverse functional factors. Some activities may have required very specific and standardized tools, which were very demanding in terms of technological investment. In contrast, other activities may have been carried out using simple artifacts with low production costs. Sometimes, these functional patterns are related to history of use and mobility, allowing us to distinguish between expedient tools made at a site and discarded after a short period of use and curated artifacts that were kept and transported. Moreover, the addition of symbolic information to some of these curated artifacts would have increased their production costs. Secondly, differences in the degree of technological investment could also have been related to people with different skills and knowledge coexisting in the same society. This distinction would have been more pronounced from the appearance of specialist production techniques, but it would always have been expressed to some extent by the contrast between experienced individuals and novices. These economic and social factors, together with other constraints related to the specific circumstances of each technical event, such as the time available for artifact production, would account for the coexistence of different levels of technological complexity within the same group and between different contemporary groups, as has been documented in ethnographic, historical, and current societies [42–45].

Revealing this intragroup variability in technological investment may be quite easy in ethnographic and historical high-resolution contexts, where establishing the contemporaneity of these different degrees of complexity is not problematic. However, this is not the case in most archeological contexts, in which the archeological assemblages are defined according to stratigraphic criteria. These assemblages should be considered palimpsests, formed by the succession of an unknown number of depositional events [46–50]. In this case, the question is whether the presence of different degrees of complexity in a lithic assemblage is the genuine expression of a real technological system or the product of mixing together artifacts from different and successive technological systems. For this reason, when appraising technical variability we should also consider the issues related to formation processes and the temporal dimension of the lithic assemblages.

The temporal integrity of the archeological record can be evaluated from burning and refitting. From several perspectives, the evidence of fire in archeological contexts can be considered a temporal marker. For instance, identifying hearths or burnt horizons can be useful for distinguishing the accumulation events, related to both anthropogenic and natural processes, that happened before and after the fire, which is particularly helpful in homogeneous sedimentary sequences. In the case of burnt artifacts, modifications produced after thermal alteration–particularly in siliceous rocks–can be identified, allowing us to differentiate the history of the artifact both prior to and after the fire damage. This has been used to identify the practice of lithic recycling [51]. Recycling of thermo-altered pebbles is evidenced at some Upper Paleolithic sites in the Côa Valley [52,53]. Lithic and bone refits can also provide data about temporal relationships [54–63]. On the one hand, there is a strong argument for the refitted items being produced during the same event and therefore being contemporaneous. On the other hand, data derived from refitting, like the scattering of the connected artifacts, the connection between activity areas, and the directionality of the movements, can yield information about the temporal dimension of the archeological assemblage. It is important to stress that, contrary to what was previously thought, the method of refitting alone cannot be used as proof of the existence of contemporaneity between different areas, especially in the case of lithic refits [64]. The temporal dimension suggested by the method can be expressed in terms of both synchrony and diachrony. Other types of information, such as the direction of the connections (unidirectional or bidirectional) and the degree of dispersion of the refitted elements can be used to demonstrate contemporaneity between areas. These two proxies–burnt artifacts and refitting–have rarely been used together in palimpsest dissection.

The aim of this paper is to analyze an expedient Upper Paleolithic assemblage to assess whether its temporal dimension can shed light on the technological variability. The selected site seems particularly suitable for this endeavor. According to previous publications [65,66], the Lagar Velho rockshelter is a high-resolution context in which one of the stratigraphic units (layer EE15, henceforth EE15) reveals a highly expedient technological behavior. Moreover, this unit is characterized by a high refitting index, particularly in a cluster centered in squares H8/I8, and a high percentage of burnt remains agglomerated in H3/H4. Here we present the lithic assemblage from the excavations at Lagar Velho that were resumed in 2018 with two main goals: firstly, to characterize the assemblage from a technological investment perspective; and secondly, to dissect the palimpsest and assess the assemblage integrity and the technological variability in its temporal framework.

## The Lagar Velho rockshelter

**Fig 1:**
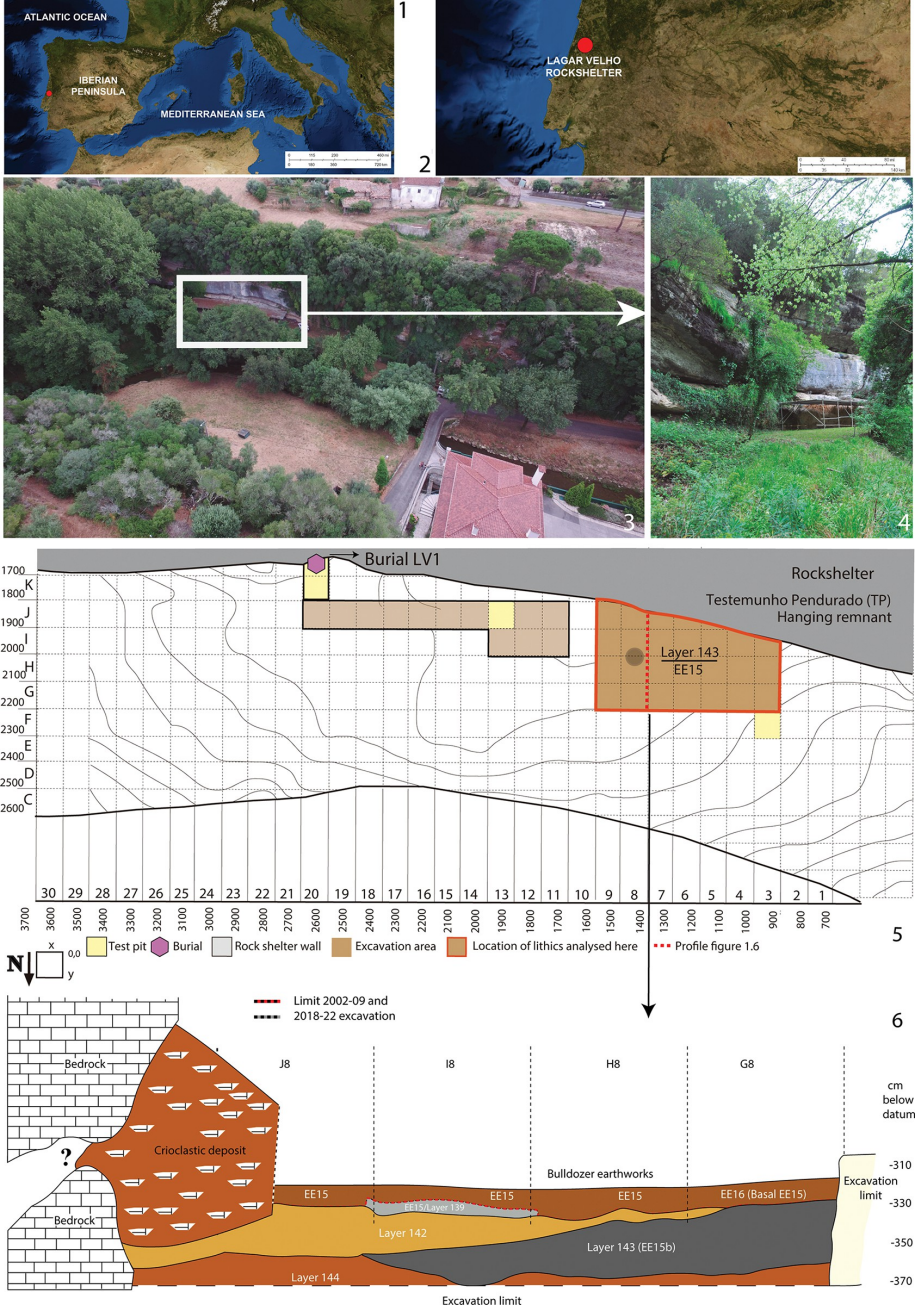
1–2. Images showing the geographical location of the Lagar Velho rockshelter (red dot). Extracted from the USGS National Map Viewer (public domain), https://www.usgs.gov/tools/national-map-viewer. 3. Aerial photo with general views of the site, view from North-northeast. 4. General view of the site, view from East. 5. Plan of the Lagar Velho rockshelter showing the location of the excavated areas. 6. Stratigraphic diagram of the layers discussed in the text.

The Lagar Velho rockshelter (Leiria, Portugal) is in the Lapedo valley, 135 km north of Lisbon (Fig 1). This valley is a deep canyon formed by the incision of the Caranguejeira creek into the Cretaceous limestones of the northern border of the Central Limestone Massif of Portuguese Estremadura. The rockshelter faces north towards Caranguejeira stream, at the base of a limestone cliff, c. 7 m above the watercourse. This site is well known in archeological research due to the 1998 discovery of a Gravettian child burial [67,68]. The ^14^C dating of the child itself failed but the ages of the archeological remains found in association with this burial dated it as being between ca. 24–25 ka BP or 29.4–27.6 ka cal. BP, taking into account the minimum and maximum ages of the interval provided by four samples that statistically do not overlap. Following this discovery, further archeological excavation was carried out between 2000 and 2009 [65]. Moreover, a rescue excavation focused on the *Hanging Remnant* (Testemunho Pendurado (TP) in Fig 1.5) took place in 2012 [68]. Finally, the current archeological project started in 2018 and is still in progress (Fig 1.5).

The archeological sequence beyond the burial includes Terminal Gravettian and Middle Solutrean occupations radiocarbon dated to between ca. 22–20 ka BP (ca. 26–24 ka cal. BP) [66]. Unfortunately, the sedimentary package that contained the remains of these occupations was largely destroyed by earthworks prior to the archeological excavations. They were only preserved in a recess of the back wall of the rockshelter (the *Hanging Remnant*). However, the underlying archeological layers were spared from destruction and one of these, EE15, was the subject of a large-scale excavation between 2000 and 2009 [65] over an area of c. 20 m^2^, comprising squares G-I/3-9 of the grid (Fig 1.5). This archeological layer, ascribed to the Late Gravettian, was in a deposit comprising essentially slope waste sediments (ms geoarcheological complex) [69] filling an elongated depression between rows 1 and 13. Many bones showing thermal damage and fire cracked blocks and cobbles have been recovered from EE15 excavation. In addition, the high index of lithic refits indicates a very good preservation of the spatial relationships. In line with this, a complex spatial pattern was proposed, including two structured fire features with different functions. Firstly, a *cuvette* structure in the western part of the excavated area (squares H3-4) was associated with large quantities of burnt bones, but very scarce lithic artifacts. In contrast, the second feature, located 3 m to the east (squares H8-I8) was practically devoid of bones but an accumulation of more than 600 lithic artifacts was found around it, allowing this feature to be characterized as a “knapping hearth”.

The technological study of the “knapping hearth” identified different lithic reduction sequences involving quartzite, quartz, and flint. Some of these were extensively reconstructed through refitting: 35% of the artifacts found in this accumulation were successfully refitted. These refitted sequences were exclusively aimed at the production of flakes and exhibited very expedient behavior. Neither predetermination nor core preparation were observed. The flake removals showed that the knappers had systematically adapted to the natural morphology of the exploited cobbles; the cortical surfaces were sometimes used as striking platforms; and the reduction sequences followed unidirectional strategies. Some large flakes produced during the initial stages of production were also exploited as cores. The vertical distribution of artifacts resulting from these reduction sequences allowed Almeida et al. [65] to suggest an inter-block diachrony in the formation of this lithic assemblage. Firstly, some imported flint tools were knapped to produce small flakes. Secondly, the main reduction sequences on quartzite cobbles and one reduction event on flint, which involved different knappers, took place. The third stage in the formation of the assemblage involved the reduction of two better-quality flint blocks. The lithic assemblage shows neither the technological nor typological characteristics typical of the Late Gravettian. Blade and bladelet production strategies are entirely absent–only two artifacts were classified as blades–and the retouched component comprises a few notches and denticulates, with a total absence of typical Upper Paleolithic tools.

The lithics analyzed in this work were recovered mainly from layers 142 and 143, targeted during the resumed excavations. Layer 143 is found in the western area of the site and has been ^14^C dated to c. 24 ka BP (29 ka cal. BP) (Table 1) [70]. It corresponds to the main occupational surface affected by fire, while layer 142 is the same layer but not thermally altered. No direct correlation between this layer and the LV1 child burial (located in the eastern part of the site) has yet been described. Finally, this study includes a few lithics recovered from a remnant excavation of EE15, labelled as layer 139 (Fig 1.6).

**Table 1:** Radiocarbon determinations based on bone samples from Lagar Velho rock shelter, layer 143. Calibration was performed by OxCal v.4.4. [71], using the IntCal20 curve [72]).

Site Sample	Provenance	Lab. #	Uncalibrated ^14^C age BP	δ 13C	Calibrated 14C age BP at 2σ
G7.10602	143 Top	OxA-X-187-12	24390±220	20,17	29112–27993
G7.10609(A)	143 Bottom	OxA-42399	24660±180	19,85	29221–28580
G7.10609(B)	143 Bottom	OxA-42400	24650±170	19,76	29230–28603

## Materials and methods

This paper centers on the lithic assemblage derived from layers 139, 142 and 143, which were excavated during the recent fieldwork project spanning from 2018 to 2021. Large set of lithics correspond to layer 143 (Table 2). Although these three layers were considered together in the analysis presented below, we should bear in mind the fact that layer 139 overlies the other two units. Excavations were authorized by the Directorate General for Cultural Heritage (DGPC) from the Portuguese Ministry of Culture, complying with all permits, legal requirements and regulations. The lithic collection is stored at the Archaeosciences Laboratory (LARC) of DGPC, in Lisbon, under the responsibility of Ana Cristina Araújo and Ana Maria Costa, both researchers at LARC. We have also revised most of the lithics from the previous (EE15) excavation stored at the same institution, although these data have not been included in this paper.

**Table 2:** Distribution of the lithics from the 2018–2021 field seasons according to layer and lithic category.

Layer	Tools	Cores	Flakes	Flake fragments	Entire cobbles	Fragmented cobbles	Cobble fragments	Total
139			3 50%	1 16.6%			2 33.3%	6
142			7 4.7%	14 9.5%	96 65.7%	4 2.7%	25 17.1%	146
143	3 0.1%	5 0.3%	91 5.3%	77 4.5%	1033 60.9%	159 9.3%	328 19.3%	1696
**Total**	**3** **0.1%**	**5** **0.2%**	**101** **5.4%**	**92** **4.9%**	**1129** **61.1%**	**163** **8.8%**	**355** **19.2%**	**1848**

To clarify the origin of the raw materials found at Lagar Velho, we sampled the cobble-bearing units surrounding the rockshelter, both from old Cenozoic deposits (Paleogene or Neogene detrital formations) and the alluvial material in the Caranguejeira creek. Five points were sampled (Table 3), two corresponding to the old Cenozoic deposits (samples 1 and 2), from each side of the Lapedo gorge, and three from the current riverbed (samples 3–5), one of which (sample 4) was from the foot of Lagar Velho rockshelter (Fig 2.4). The sampling method consisted of locating an area in which the cobble deposits were exposed and then delimiting a surface of 1 m^2^, from which a minimum of 50 cobbles were collected. This procedure follows the sampling methods used in hydraulic engineering [e.g., 73]. In the cases where this number could not be reached in 1 m^2^, the collection radius was expanded until a maximum radius of 5 m. This was the case of the two samples from the old Cenozoic deposits. The selection of the areas to be sampled was based on the visual inspection of the cobble-bearing formations, selecting those with the highest density of cobbles.

**Fig 2:**
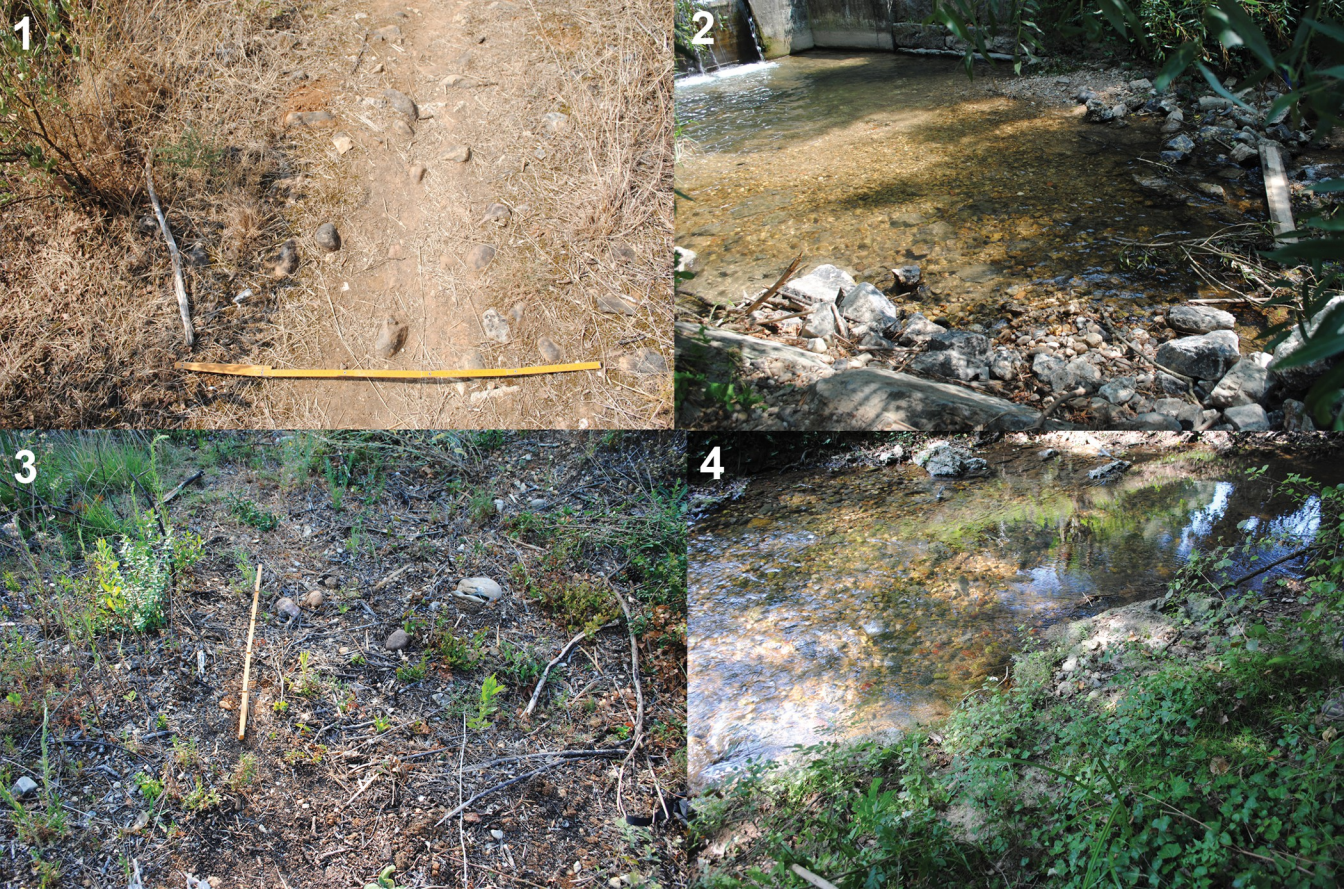
Images of four of the five locations selected for sampling, corresponding to both the old Cenozoic deposits (1, 3) and the current riverbed (2, 4).

**Table 3:** Geographic coordinates and geological context of the cobble sampling locations. Coordinate system: WGS94.

Reference	Coordinates		Geological context
	Latitude	Longitude	
Sample 1	39.757	-8.7311	Paleogene and Neogene detrital formations
Sample 2	39.7581	-8.7332	Paleogene and Neogene detrital formations
Sample 3	39.7607	-8.7311	Present-day riverbed deposits
Sample 4	39.7559	-8.734	Present-day riverbed deposits
Sample 5	39.7555	-8.7419	Present-day riverbed deposits

Most of the assemblage is made up of entire or broken cobbles. We distinguished three different categories for these elements: entire cobbles, fragmented cobbles–broken cobbles for which the original size and shape can be estimated–and cobble fragments–the original size and shape cannot be estimated. The study of the elements showing knapping features is based on attribute analysis specific to each of the main artifact categories: cores, flakes, and retouched pieces. Only artifacts larger than 1 cm were considered in this analysis. Length, width, thickness, and weight were recorded for all lithics and five size categories were created taking into account length (l) and width (w): very small (<500 mm^2^), small (500–1000 mm^2^), medium (1000–1500 mm^2^), large (1500–2000 mm^2^), and very large (>2000 mm^2^). Moreover, the elongation index was calculated for the complete flakes (l/w). Artifacts with an elongation index greater than 2 were considered to be laminar products. Among the laminar artifacts, those narrower than 12 mm were classified as bladelets.

For the core analysis, we considered the structural attributes: number and location of striking platforms, number and characteristics of the flaking surfaces, hierarchization of the flaking surfaces, and reduction degree. In terms of the debitage products, we recorded different attributes for the striking platform (cortex cover, platform type, and preparation), dorsal face (cortex cover), and ventral face (bulb of percussion type and ventral curvature). Finally, the retouched artifacts were analyzed and classified according to the Laplace method [74]. This method is based on an attribute analysis in which different characteristics of the retouch are considered (location, angle, depth, direction, and form). From these attributes, different primary types and typological groups (e.g., sidescrapers, denticulates, endscrapers, backed blades…) are defined.

We made a first attempt at the spatial distribution of the lithic remains. All the lithics remains were individually plotted, but the cobbles were exclusively three-dimensionally plotted during the 2018 campaign and subsequently bagged on meter square. We therefore calculated coordinates at random in order to obtain a broader view of the spatial pattern, paying special attention to the burnt lithics. Kernel Density Estimation was applied to produce an image of the spatial distribution of the lithics. However, we have not included the lithics with random coordinates in the vertical projections. The software used for the spatial analysis is QGIS version 3.18.3.

A refitting program was carried out, in which the elements from both EE15/139 and 142/143 were considered. However, since the lithics from the EE15 collection had already been the object of an extensive refitting program, our main interest in these artifacts was to find connections with the elements from layers 142/143 in order to refine the stratigraphic correlations. The lithics from EE15 have only been considered in refitting, but their analysis is not included in this paper.

Refitting was carried out in two phases: i) during the excavation campaigns, using the field laboratory and ii) during a campaign carried out specifically for this purpose at the DGPC’s Archaeosciences Laboratory in Lisbon, where the Lagar Velho archaeological collections are stored. In total, about 200 hours were invested in searching for refits. The refitting procedure started with the segregation of lithics according to the main lithological categories (quartzite, quartz, sandstone…). Next, the lithic remains of each of these categories were grouped according to their macroscopic characteristics and each group was extended on a table. Considering the particular characteristics of the Lagar Velho lithic assemblage, we have distinguished the following types of refits:

- Fragmented cobbles. This type of refit corresponds to the connection of fragments from broken cobbles for which the cause of the breakage has not been determined.
- Breakage. Connection between indeterminate fragments.
- Isolated removals. Connection between cobbles and isolated removals detached from them by percussion.
- Knapping. Connection between the products derived from core reduction or tool manufacture sequences.

In the case of knapping refits, connection lines have been calculated considering the chronological order of the removals [75]. For breakage, no chronological sequence could be established so the connection lines link the contacting surfaces. When tridimensional coordinates were available, we calculated the length and orientation of the connection lines.

In our study, we paid special attention to the refitting of burnt lithics, since these are particularly informative in terms of the temporal dimension of the archeological assemblage. For these refits, two situations with quite different temporal meanings can be envisaged:

- All the artifacts making up the refit are burnt and exhibit the same degree of burning. These refits are the least diagnostic for establishing the temporal relationship between the breakage and the thermal damage. Although breakage during or after burning seems the most likely explanation, the opposite–breakage before burning–cannot be entirely rule out, especially if the fragments remained close to one another after breakage.
- Temporal patterns are more evident when both burnt and unburnt lithics are included in the refit or the refitted element show different degrees of thermal damage. In these cases, it seems clear that the event producing the breakage was prior to the exposure to fire. Moreover, it means that the fragments were separated enough to be differentially affected by fire.

## Results

### General patterns: Lithic categories, raw materials and taphonomy

At first glance, it was evident that two different components could be recognized in this assemblage. On the one hand, there was a huge number of cobbles and cobble fragments, most of which presented no clear evidence of modification or use discernable at naked eye. This component makes up the majority of the 142/143 collection (89.1%). On the other hand, there were artifacts that showed attributes normally related to knapping processes, but this component is much rarer. Most of these purported artifacts are flakes and flake fragments. Cores and particularly tools are scarce.

Both components described above are characterized by the predominance of quartzite (46.4%) and quartz (33.8%) (Table 4). This is even more evident if we consider exclusively the artifacts potentially related to knapping, although in this case quartz (54.4%) is more common than quartzite (35.6%). Among the knapping products, we would like to highlight the small percentage of flint artifacts (5.4%). In the cobble assemblage, besides quartzite and quartz, only sandstone, porphyry, and limestone are present in significant percentages. Except for flint, all these rocks are available in the immediate surroundings of the site. As already mentioned, quartzite and quartz are particularly abundant in the alluvial formations of Caranguejeira creek and in the old Cenozoic deposits above the site. Flint sources are not far from the rockshelter. Cenomanian flint outcrops have been well documented in the Leiria region [76] and the nearest flint sources have been identified only 3.5 km away from Lagar Velho, in the Ribeira das Chitas valley [65]. Additional flint outcrops are located in Rio Maior and Caxarias, the latter being less than 20 km from the Lapedo valley [65,77]. However, no flint nodules were found during the surveys of the secondary formations around the site.

**Table 4:** Distribution of the lithics according to raw material and lithic category.

Raw material	Tools	Cores	Flakes	Flake fragments	Entire cobbles	Fragmented cobbles	Cobble fragments	Total
Sandstone			4 4%	1 1.1%	137 12.1%	39 23.9%	42 11.8%	223 12%
Limestone					39 3.4%	3 1.8%	16 4.5%	58 3.1%
Rock crystal				1 1.1%				1 0.05%
Quartzite	3 100%	3 60%	43 42.6%	23 25%	596 52.8%	63 38.6%	128 36%	859 46.5%
Quartz		2 40%	47 46.5%	61 66.3%	308 27.3%	51 31.3%	156 43.9%	625 33.8%
Indeterminate					4 0.3%	2 1.2%	1 0.3%	7 0.3%
Metamorphic					5 0.4%	3 1.8%	4 1.1%	12 0.6%
Porphyry			1 1%		40 35.4%	2 1.2%	8 2.2%	51 2.7%
Flint			6 5.9%	6 6.5%				12 0.6%
**Total**	**3**	**5**	**101**	**92**	**1129**	**163**	**355**	**1848**

A significant proportion of the lithics (30.3%) exhibits macroscopic signs of thermal damage, which was expected given the abundant evidence of fire in layer 143 (Table 5). If we look at the two above-mentioned components, it seems that the lithics showing attributes consistent with knapping were less affected by fire, since their percentage of thermal damage (16.9%) is lower than that shown by the cobble assemblage (31.9%). There are not significant differences between layers 142 and 143 in terms of the percentage of burnt lithics (35.6% and 30%, respectively). There are not lithics with thermal damage in layer 139, although we must remember the small number of lithics assigned to this layer (only 6).

**Table 5:** Distribution and percentages of burnt lithics according to layer and lithic category.

Layer	Tools	Cores	Flakes	Flake fragments	Entire cobbles	Fragmented cobbles	Cobble fragments	Total
142			2 28.5%	3 21.4%	38 39.5%	3 75%	6 24%	52 35.6%
143	1 33.3%	1 20%	18 19.7%	10 12.9%	319 30.8%	57 35.8%	103 31.4%	509 30%
**Total**	**1** **33.3%**	**1** **20%**	**20** **19.8%**	**12** **13%**	**357** **31.6%**	**60** **36.8%**	**109** **30.7%**	**561** **30.3%**

As can be seen in Fig 3.1, the highest densities of lithic remains were found in the outermost parts of the excavated area, in squares G7 (23.6% of the lithics), G8 (17.2%), and G5 (12.4%). The number of lithics decreases towards the inner part of the rockshelter. The distribution of the burnt lithics exhibits the same pattern (Fig 3.2). Likewise, the vertical projection of burnt and unburnt lithics in row G (Fig 3.3) suggests similar distributions. It therefore appears that there is no spatial segregation of the lithics based on thermal damage, neither in the horizontal nor in the vertical dimension.

**Fig 3:**
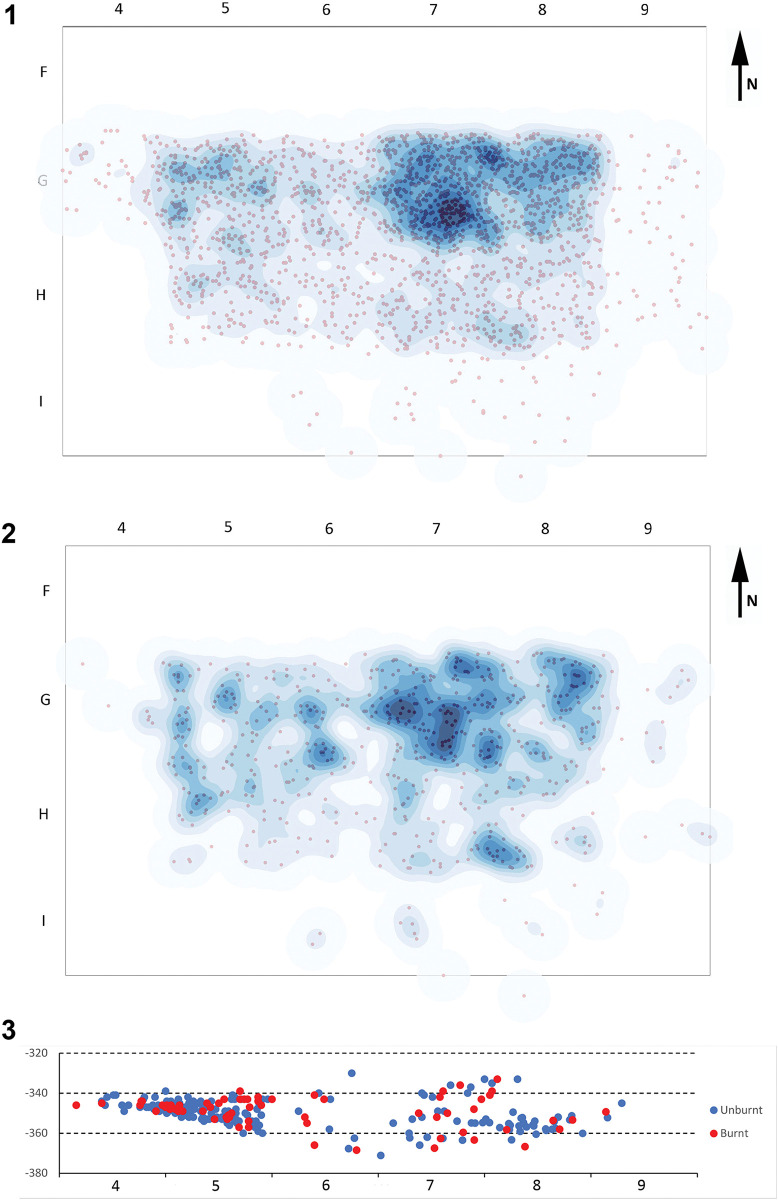
Spatial distribution and Kernel density estimation of all lithics (1) and burnt lithics (2). Both the lithics mapped in the field and those with random coordinates are represented in these maps. 3. Vertical projection of burnt and unburnt lithics in row G. In this case, only the lithics mapped during fieldwork are included. The differences in density between columns 4–5 and columns 6–9 are related to changes in the mapping criteria. The lithics represented in this figure correspond to the 2018–2021 excavation (layers 139, 142 and 143). The lithic remains from EE15 excavation are not included.

### The cobble assemblage

With regard to cobbles, the first issue that must be addressed involves the formation processes that could explain their accumulation at the site. They are allochthonous inputs, but the agent responsible for their introduction into the site is not entirely clear. Apart from the flint, all the raw materials documented in the archeological assemblage are represented in the natural samples collected in our geological survey. Likewise, quartzite and quartz are dominant at all the sampled points. Just as in the archeological assemblage, quartzite is the most widely represented in all the natural samples, except Sample 1, which corresponds to the old Cenozoic deposits on the left-hand margin of Caranguejeira creek, just above the rockshelter. In this sample, quartz is the predominant material. Sandstone, limestone, and porphyry cobbles were also documented in most samples, apart from Sample 3, where limestone and porphyry cobbles are absent. In terms of cobble weight, there are some interesting differences between the archeological assemblage and the natural samples. In the former, most of the cobbles (85.9%) weigh less than 300 g and very few weigh more than 500 g (1.6%); no entire cobble weighs more than 700 g. In contrast, in the natural samples, there are significant numbers of quartz, quartzite, sandstone, and porphyry cobbles weighing more than 500 g; they represent between 48% (Sample 2) and 6.5% (Sample 3). In the Caranguejeira sample closest to the site (Sample 4), they account for 18.4% of the total. Moreover, at all the sampled points some very large boulders, weighing more than 1 kg, were found. It therefore seems that the archeological assemblage exhibits a volumetric selection of cobbles, where the larger sizes are absent.

**Fig 4:**
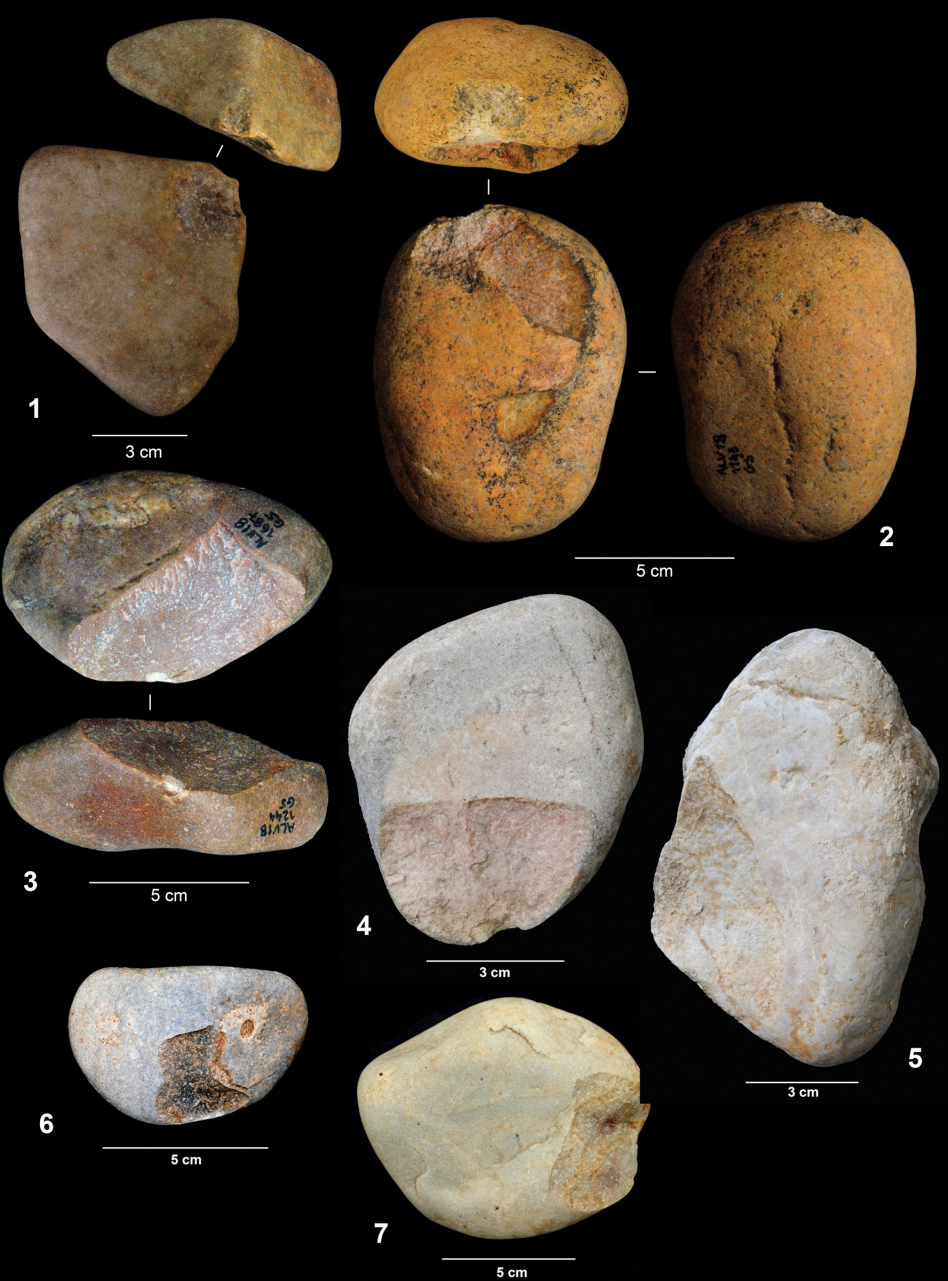
Cobbles showing isolated removals.

According to their completeness, we were able to distinguish three different categories in the cobble assemblage: entire cobbles, fragmented cobbles for which the form and size can still be estimated, and cobble fragments whose original size and shape is unknown. The first category is clearly dominant and represents 68.5% of the cobble assemblage and 60.6% of all the lithics found in layers 142/143 and 139. Although most of the elements included in this assemblage do not show any evidence of modification besides breakage, some of them (N = 100, 10.6% of the entire cobbles, and N = 24, 20.1% of the fragmented cobbles) exhibit removals detached by percussion (Fig 4). In most cases (62.6%) there is only one isolated removal, but some cobbles show two (31.6%) or three (5.7%) detachments. It is worth stressing that none of them present other possible evidence of use in addition to these removals. Although we have included these lithics in the cobble assemblage, they could also be considered part of the knapped assemblage, since they exhibit attributes consistent with intentional knapping. In any case, these cobbles with removals express the complexity of the Lagar Velho formation processes and, as we will discuss later, the difficulties in discerning natural inputs from the products resulting from human activities.

### The knapped assemblage

As indicated previously, the artifacts yielding attributes consistent with knapping comprise just a small part (almost 11%) of the lithic assemblage. Most of these artifacts correspond to flake and flake fragments derived from the reduction of quartz and quartzite cobbles. As we will see later, the reduction of some of these cobbles on the site is evidenced through refits. However, 24 flakes (23.7%) are whole cortical products that can be related to the single removals from cobbles referred to in the previous section. As we will see later, in some cases this has been confirmed by refits. All the sandstone flakes correspond to this kind of artifact, reinforcing the preferential use of quartz and quartzite for flake production. It seems likely, therefore, that at least one part of the knapped assemblage corresponds to the process responsible for these single removals.

**Fig 5:**
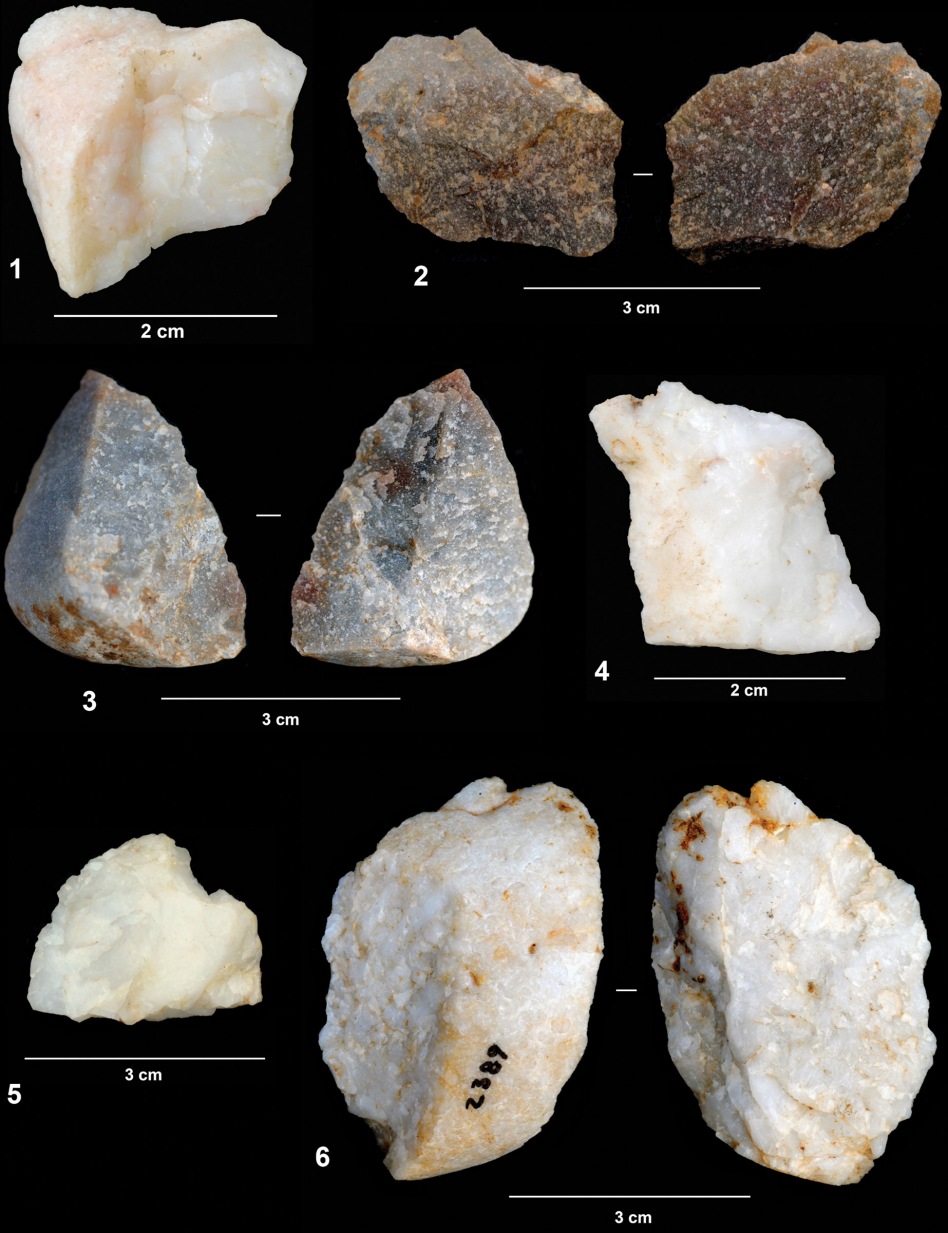
Quartz and quartzite flakes.

From the volumetric perspective, very small and small flakes are predominant (accounting for 72.7% of the flakes). Only three very large blanks have been found. With the exception of two flint bladelets, no evidence of laminar production has been identified. One of the most outstanding characteristics of the flake assemblage is the high cortex index. More than 73% of the flakes exhibit cortical surfaces (partial or total), indicating that products derived from the first stage of reduction sequences are in the majority (Fig 5). This is also evident if we look at the striking platforms, since these are cortical in 54.5% of the flakes. Although these percentages may be somewhat overstated due to the presence of the above-mentioned single removals, they are related to two basic characteristics of the knapping strategies documented from the assemblage: first, the absence of core preparation and the use of cortical surfaces as striking platforms; second, the presence of short reduction sequences, in which only the superficial part of the volumes is removed. This is particularly evident in the refitted sequences that we will describe later.

The analysis results for the five cores recorded in the 142/143 and 139 collection is consistent with the data provided by the flakes. The cores show the preferential use of unidirectional strategies aimed at flake production. They show no evidence of blade or bladelet production. In most cases, cortical surfaces were used as striking platforms, but there are two cores in which flakes were detached from fracture planes. In no case has preparation of the striking platforms or the flaking surfaces been observed. Reduction sequences were relatively short and only 9–10 products were obtained from the longest. Consequently, totally or partially cortical products tend to dominate. In fact, some cores displaying very few removals blur the boundary between these and the cobbles with single detachments described previously.

Three of the cores were largely refitted, enabling a better understanding of the reduction sequence applied in flake production. Refit 31 consists of nine quartzite artifacts (the core, seven flakes and one flake fragment), most of which were found scattered across squares G5 and H5 (Fig 6.1). It exhibits a clear unidirectional strategy on a quartzite cobble of flat-convex section. From a cortical striking platform, a series of flakes were detached from one of the cobble sides. The abandoned core presents a chopper-like morphology. Six of the eight refitted products have cortex on their dorsal faces and all exhibit cortical striking platforms.

**Fig 6:**
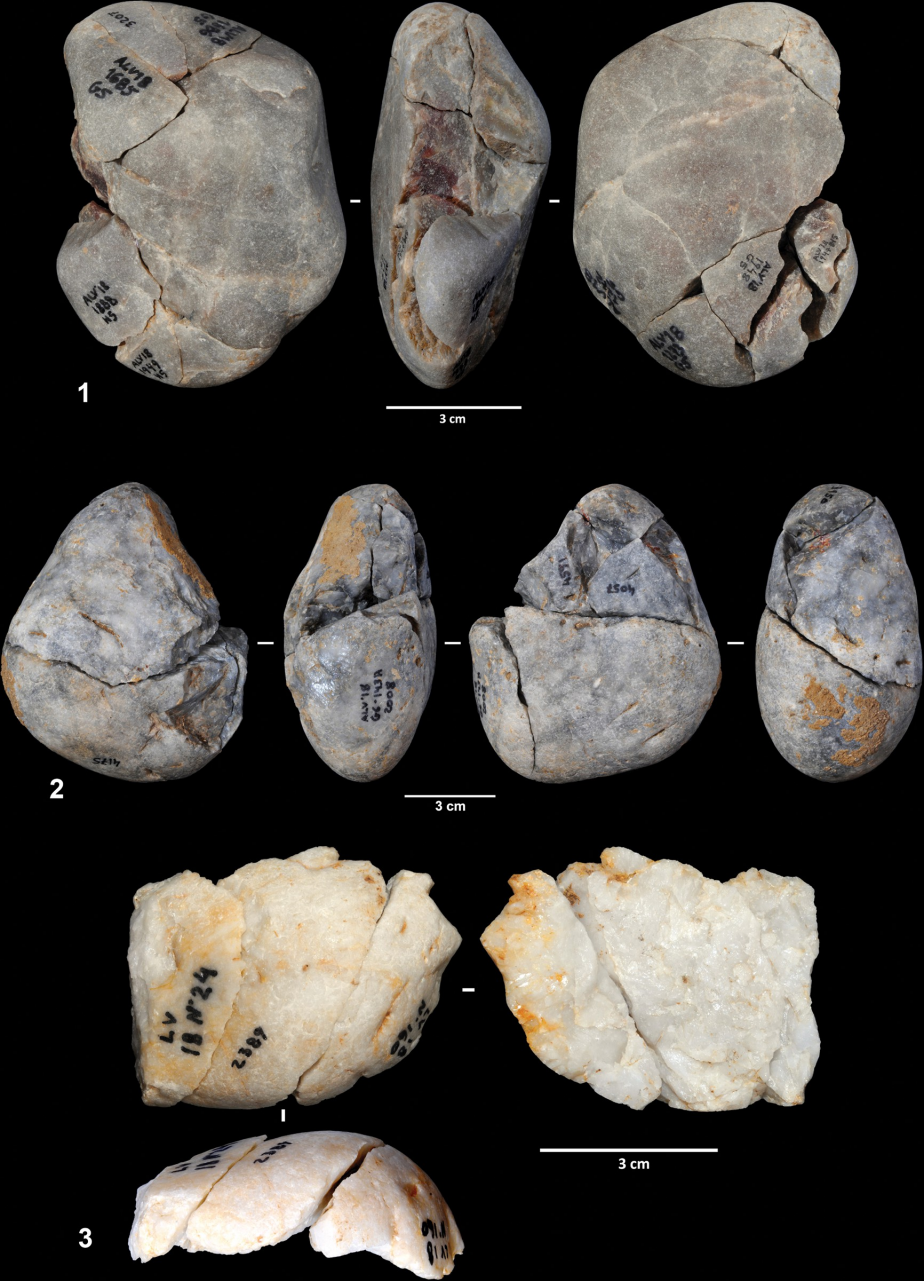
Reduction sequences on quartzite and quartz cobbles. 1. Refit 31. 2. Refit 56. 3. Refit 73.

Refit 56 is made up of six artifacts and corresponds to the exploitation of a gray quartz cobble (Fig 6.2). The cobble was broken into two fragments that were exploited independently. The artifacts were scattered across squares G6, G7, and H7, although most of the pieces were clustered in square G7. One of the cores was found in layer EE15, one of the direct connections between the two excavation phases. Three flakes were detached from one of the cobble fragments, although only one of these could be physically refitted. The fracture surface was used as a striking platform, and the volume was exploited following a unidirectional strategy. The second fragment was exploited from two different striking platforms, the fracture surface, and a lateral natural surface. Three flakes were detached from this second core. All the products have cortical dorsal faces.

Ten artifacts (one core and nine products) were connected in refit 41, the exploitation of a quartzite cobble of irregular morphology (Fig 7.1). Seven of the eight artifacts were clustered in square G8, with the remaining lithics found in squares G7 and H8. Flakes were obtained from three different striking platforms. First, four flakes were detached from a natural surface. Then, this flaking surface was used as a striking platform from which two entirely cortical flakes were produced. Two additional flakes were detached from a third striking platform located on the opposite side of the cobble; however, the chronological relationship between this and the other two platforms cannot be established. As in the previous cases, most knapping products are totally or partially cortical on both the dorsal faces and the striking platforms.

**Fig 7:**
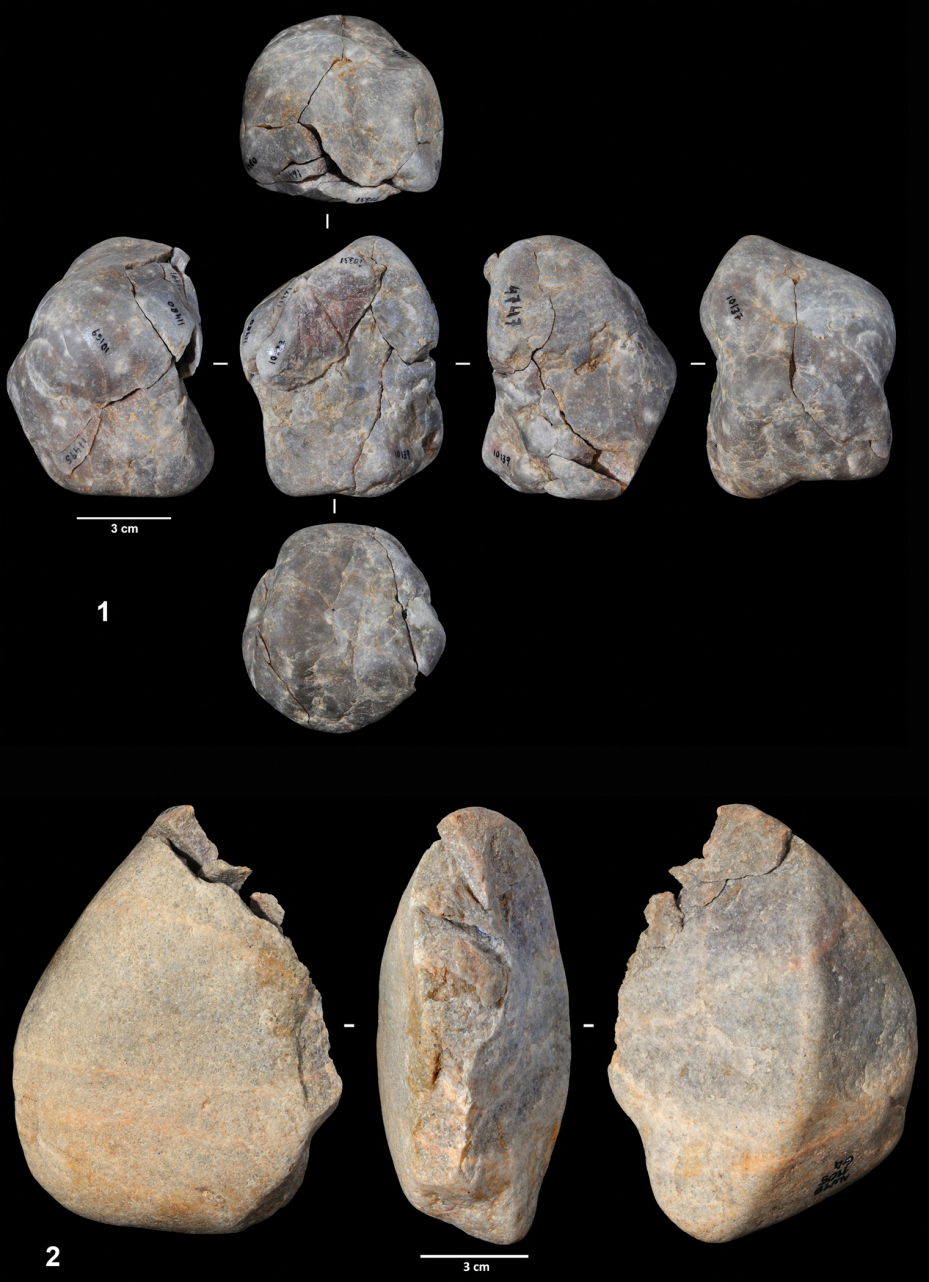
Reduction sequence on a quartzite cobble (refit 41). 2. Refit of a worked cobble sequence.

**Fig 8:**
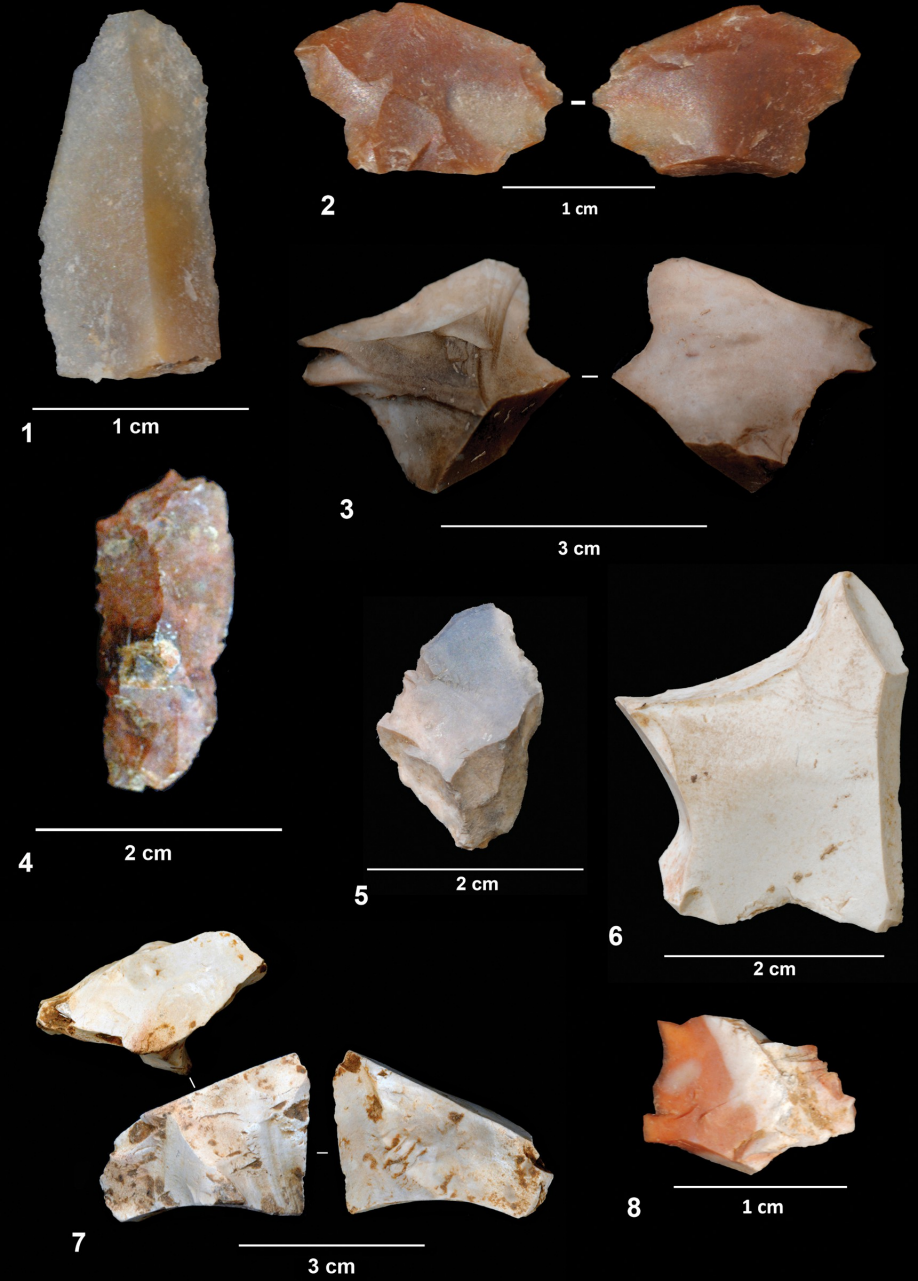
Flint artifacts.

Flint artifacts recorded from layers 142/143 are very scarce. Only thirteen flint artifacts have been identified, flakes and flake fragments, all of which are very small and small (Fig 8). According to their raw material type and provenance, one flake and one flake fragment from layer 142 found in square I8 (Fig 8.2 and 8.8) could be related to one of the reduction sequences from the “knapping hearth” (the so called SVZ block) [65], but refitting was unsuccessful. Although layer 142 is stratigraphically below EE15, in some areas the two layers present the same depth due to the sediment surface morphology (Fig 1). The remaining flint artifacts cannot be related to reduction sequences carried out at the site and must therefore have been moved into the excavated area as single items from outside. The presence of two bladelets is particularly noteworthy, as these are the only laminar products identified from the 142/143 assemblage (Fig 8.1 and 8.4). These two artifacts were classified as bladelets according to their elongation index, but they show no other features commonly associated with bladelet production methods, such as the presence of parallel scars from previous removals. Unlike the quartzite and quartz artifacts, few cortical surfaces have been identified among the flint objects and two of them show evidence of thermal damage.

**Fig 9:**
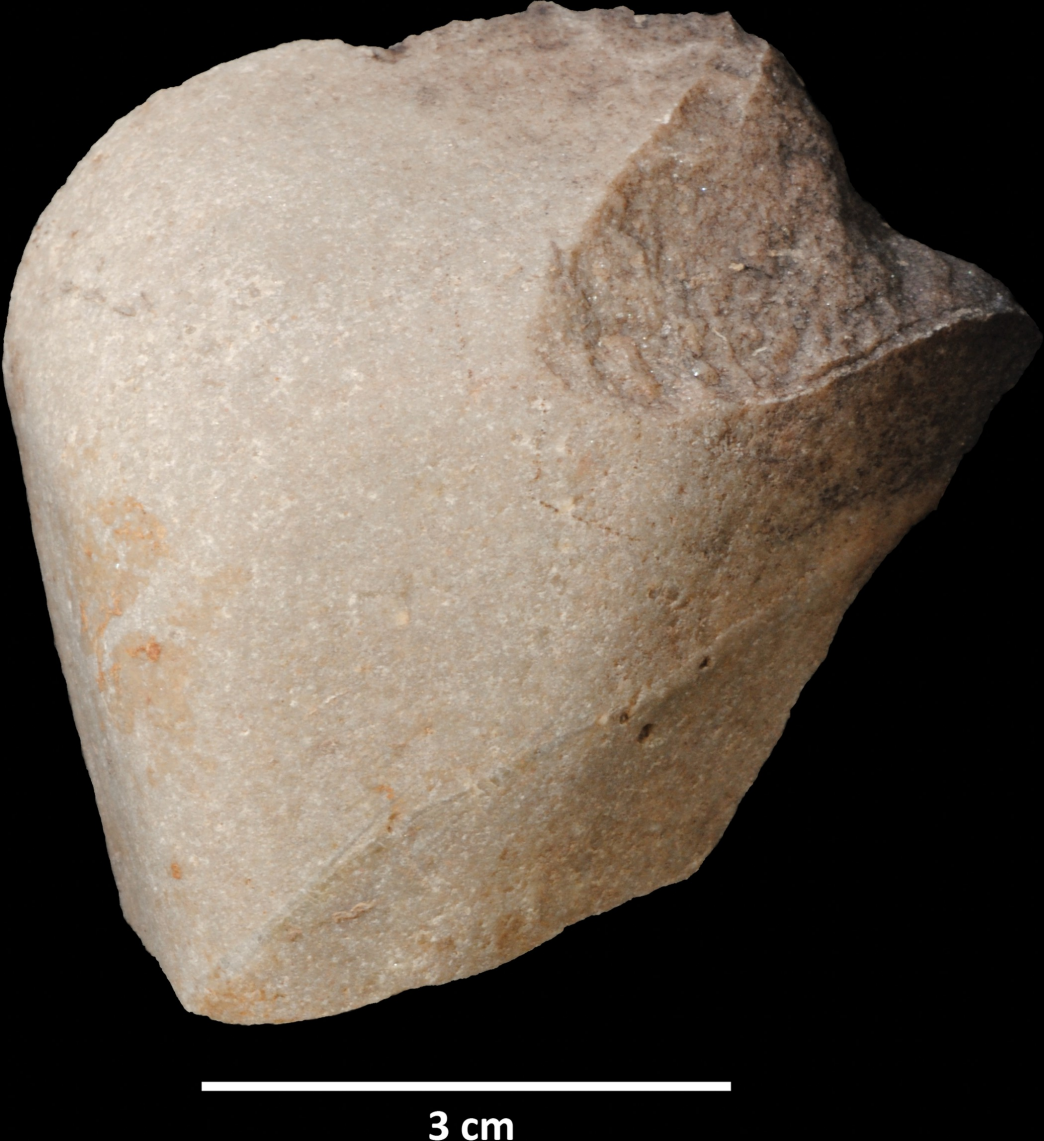
Lithic classified as a retouched artifact.

Only three artifacts have been classified as retouched pieces, which agrees with the small number of retouched tools found in the previous excavation (layer EE15). Two of these were made on quartzite flakes. The first shows a notch on the distal side of the blank (Fig 9), while the second presents a denticulated retouch on the left edge of a cortical flake. The third tool is a quartzite cobble with a chopper-like morphology (Fig 7.2). Even though we have included this artifact in the tool category, it could also have been considered a core reduction sequence aimed at the production of small flakes. It exhibits a very robust trihedral at the intersection between the retouched left side and the cortical right side. At least five different removals have been identified, three of which have been physically refitted with the cobble, indicating that the artifact was manufactured at the site. Spatially, the refitted flakes were found in squares G6 and H6, slightly separated from the tool/core, which was recovered in square G4. However, this distance is consistent with the dispersal range of knapping and intentional movement cannot therefore be determined.

### Refitting

**Fig 10:**
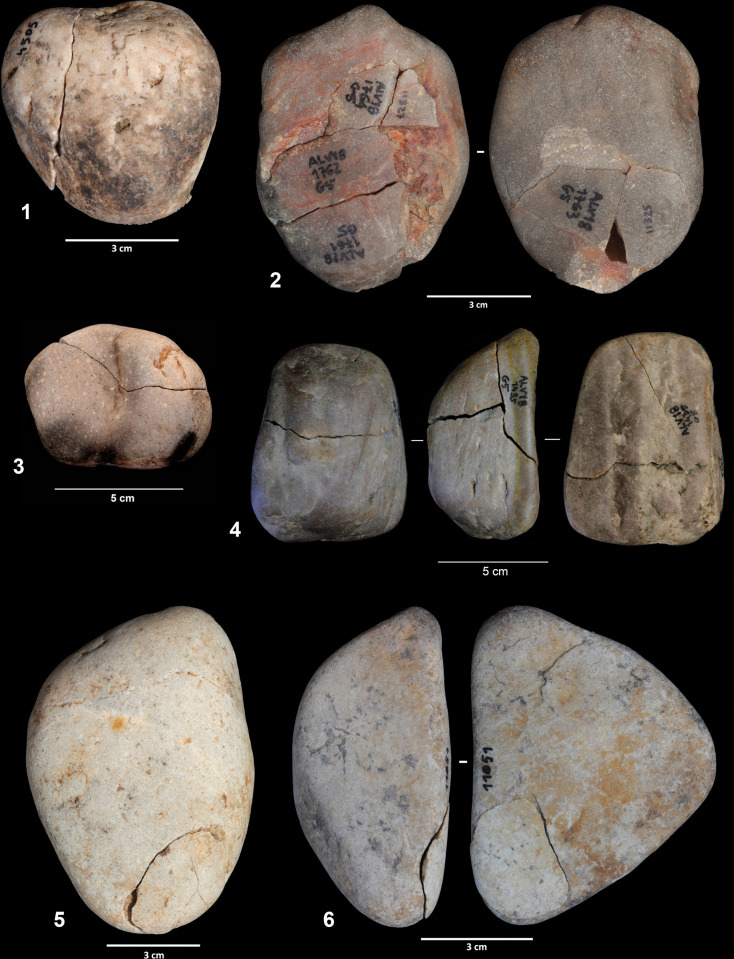
Refits of fragmented cobbles (1–4) and cobbles with isolated removals (5–6).

We have refitted 257 lithics from layer 142/143 (Fig 10). This represents 13.9% of the whole lithic assemblage, but 31.5% if we exclude the entire cobbles. As far as the stratigraphic units are concerned, most refits connect elements found in the same layer, particularly layer 143. This demonstrates that the lithics form a coherent stratigraphic assemblage, in which post-depositional displacements are uncommon. We found five refits between layers 143 and 142, the latter located above the former. Layer 142 corresponds to an area with less human activity (lithics and bones). Connections between the two layers are probably related to natural sedimentation and the short movements of artefacts along voids between blocks or mass displacement during infilling.

Moreover, eighteen lithics from previous excavations were refitted with items from the current excavations. The connections shed light on the sedimentation processes and support the inferences drawn from the field data. When analyzed in detail, ten refits are between the same layers which were assigned different names in previous and current fieldwork campaigns. Seven refits are from the very top of layer 143 (excavated between 2000 and 2009 and named EE15b) and 143, and three are from EE15 and 139. Four linked the basal part of EE15 and the top of 142/143, which could be explained by the proximity between the two layers in relation to natural sedimentation dynamics. Finally, only three refits linked layers EE15 and 143, which are statistically insignificant (1%) in terms of vertical distribution.

We found 102 refits, many of them (n = 69, 67.6%) made up of only two lithics, although some of the refits include seven, nine and ten artifacts. The latter correspond to the core reduction sequences described above. In Table 6, we can see the distribution of the refits according to the number of lithics and the type of refitting. Most refits correspond to fragmented cobbles, followed by the refitting of isolated removals and knapping sequences. Of the latter, practically all are core reduction sequences, with the possible exception of the refit corresponding to the manufacture of the chopper-like artifact.

**Table 6:** Distribution of refits according to the number of elements and the nature of refitting.

Number of elements	Fragmented cobbles	Breakage	Isolated removals	Knapping	Total
2	45	6	18		69
3	11	1	2	1	15
4	10			1	11
5	2				2
6	1			1	2
7	1				1
9				1	1
10				1	1
**Total**	**70**	**7**	**20**	**5**	**102**

**Fig 11:**
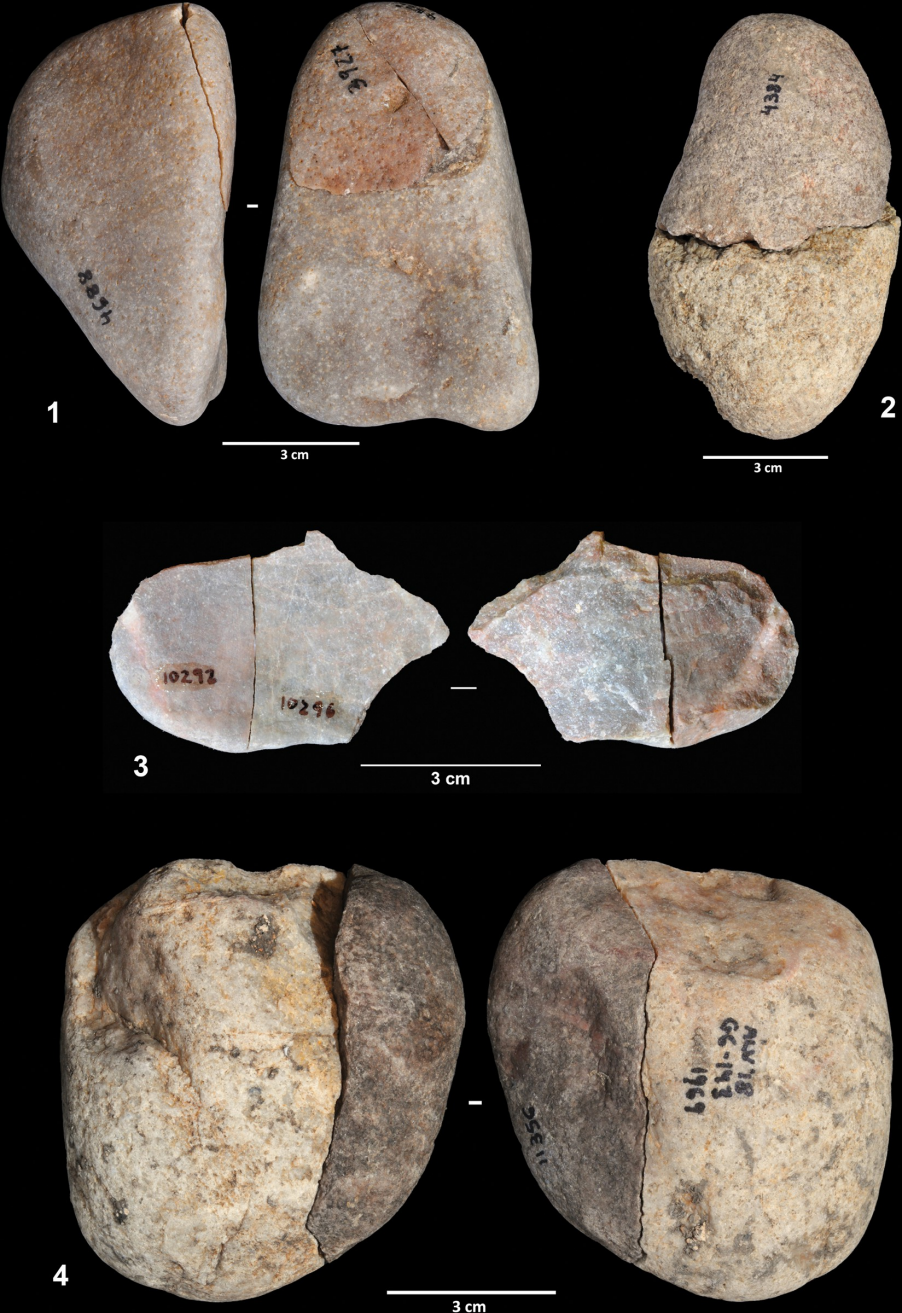
Refits including burnt and unburnt lithics.

**Fig 12:**
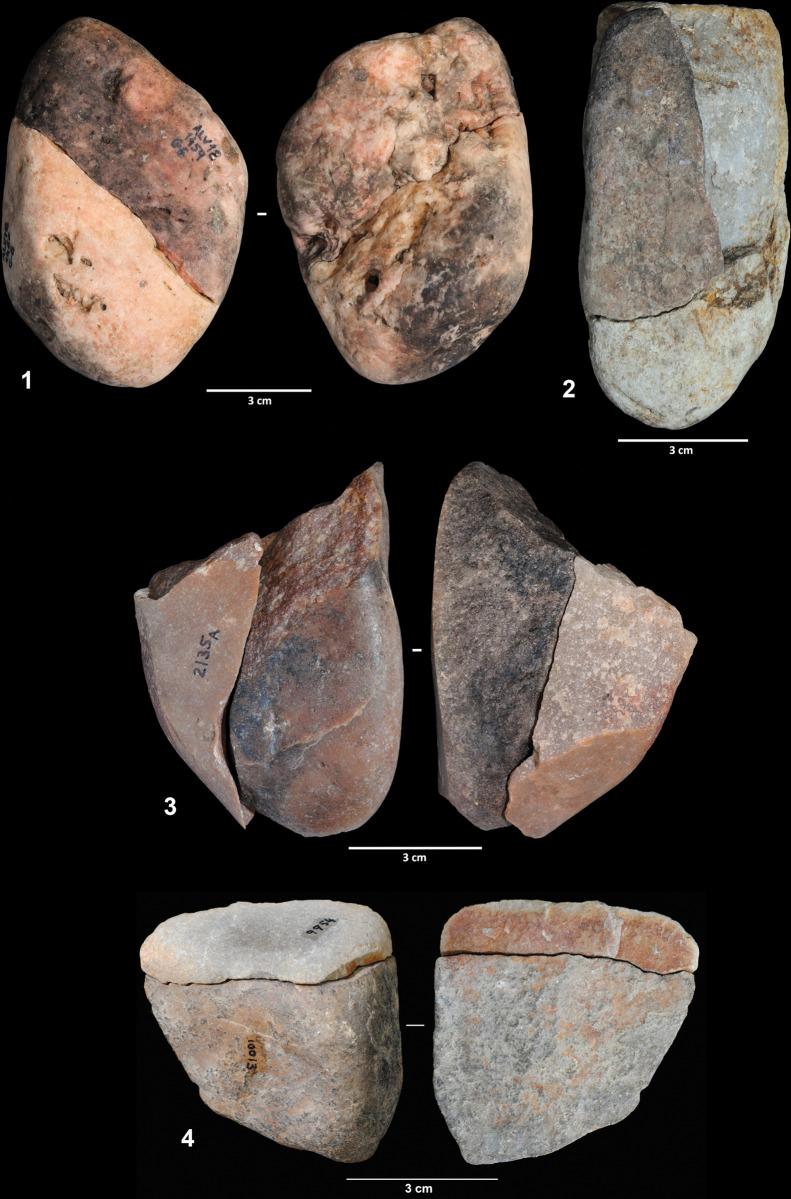
Refits including burnt and unburnt lithics.

Thermal damage is also evident among the refitted lithics, although the percentage of burnt items (27.6%) is somewhat lower than that of the entire lithic assemblage. These refitted burnt lithics are particularly interesting, since they can potentially be used to identify the succession of the various events involved in the formation of the archeological assemblage. Of the 45 refits including elements with thermal damage, in 17 cases all the lithics making up the refit are burned and show the same degree of damage. Breakage after or during burning therefore seems the most likely explanation. However, there are also 28 refits that connect burned and unburned lithics, suggesting that the breakage was produced before some lithics were affected by fire (Figs 11 and 12). There are 34 refits of fragmented cobbles with burnt lithics, 12 of them broken after/during burning and 21 beforehand. In addition, two breakage events were recorded in one case, one of which was prior to burning and the other subsequent to it. Breakage before burning is also predominant in the isolated removal refits (five out of seven cases). Of the four breakage refits including burnt lithics, three show fragmentation after/during thermal alteration and one before it. It must be highlighted that none of the knapping refits include burnt artifacts.

These refits result in 173 connection lines. Since not all the lithic remains involved tridimensional piece plotting, we were able to calculate the exact length of 43 of these connection lines (Fig 13.1). Their mean length is 62.09 cm and there are no significant differences according to the type of refit (F = .98, p = .41). Of these 43 lines, ten are longer than a meter and only one of them exceeds two meters. There is no correlation between the weight of the lithics and the length of the connection lines (r = -.011, p = .929). For the other connections, we only have a reference to the square. Connections between non-adjacent squares were established in four of these cases. Of these, three have a maximum length of between 3 and 4 m, and one could exceed 5 m (between squares G4 and G8). Although the relatively small dimension of the excavated surface limits the refit distances, the clear predominance of short connections suggests that most lithics have not experienced significant horizontal movement and therefore the spatial relationships between the remains are relatively well preserved. However, the orientation of the connection lines reveals a preferential WNW-ESE pattern (Fig 13.2). The Chi-square test indicates that this orientation pattern is statistically significant (X^2^ = 16.3; df = 5; p = .006). The general trend of the Lagar Velho layers is towards the south and the topographic morphology of the site is complex in this sector: the Gravettian paleosurface seems to correspond to an elongated depression into which large boulders collapsed from both the back wall and overhangs, creating a protected and confined area that preserved the sedimentary infill and archaeological occupations. With regard to vertical movements, most connections exhibit very short distances, of less than 5 cm (Fig 13.3). However, there are three cases in which the vertical separation between the refitted lithics exceeds 10 cm, with a maximum length of 16 cm for a connection between squares G5 and H6. For this reason, a generalized disturbance of the deposit seems unlikely, but some specific vertical movements cannot be ruled out.

**Fig 13:**
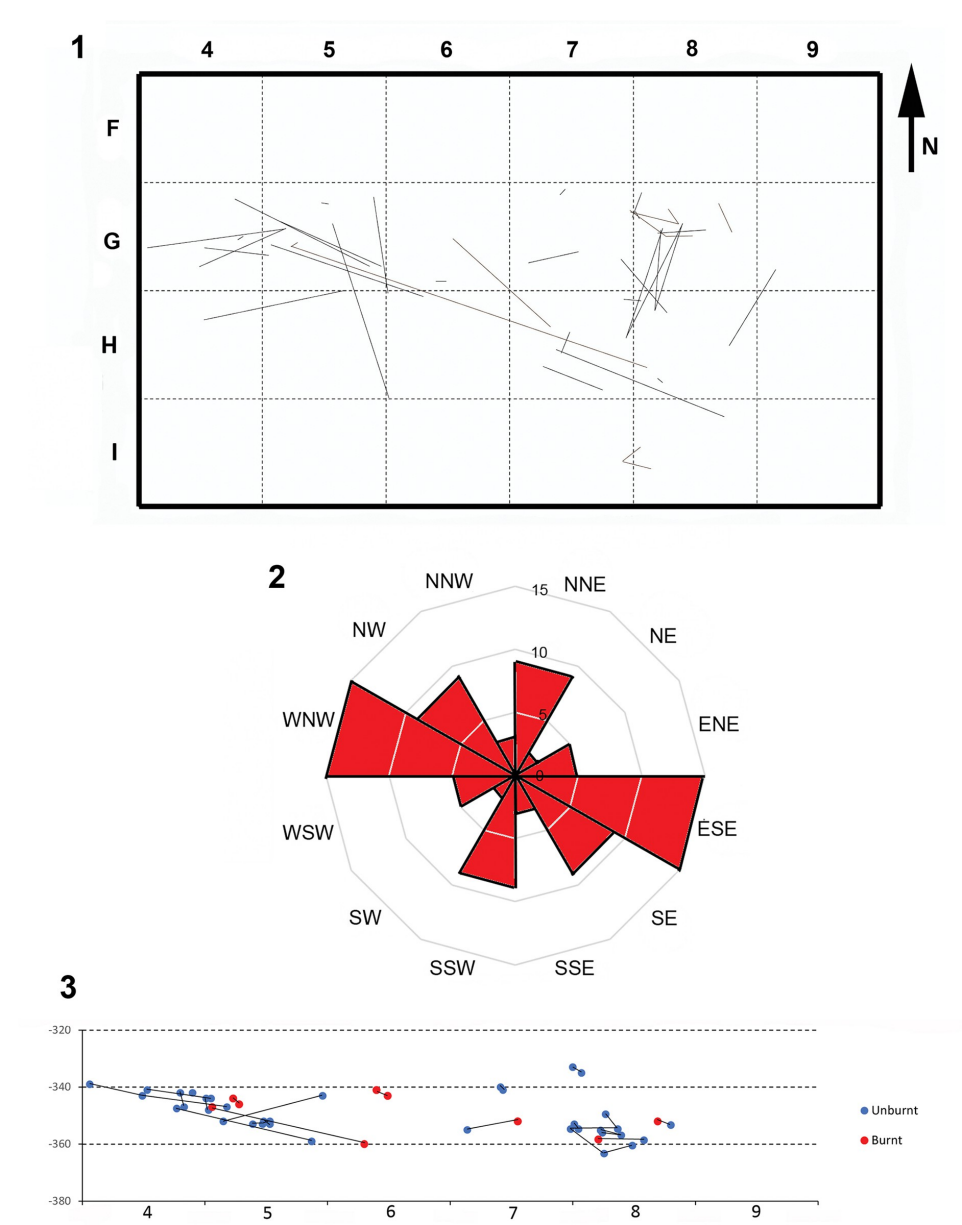
1. Map of connection-lines. 2. Rose diagram of connection-line orientation. 3. Vertical projection of the connection lines in row G ((altitude in m below datum).

## Discussion

### The cobble assemblage

The first issue that must be discussed is the origin of the large accumulation of cobbles in the assemblage. One of the most outstanding features documented at Lagar Velho is the high density of cobbles and fragments derived from the breakage of cobbles. This component is clearly predominant over artifacts derived from knapping in layer 142/143 (89.1%). In the assemblage from the EE15 excavation, the knapping products are mostly associated with the “knapping hearth”, but the cobble component was also predominant over the rest of the excavated area (NR = 354 cobbles and 45 fragmented cobbles representing 42% of the EE15 assemblage [65]). As these cobbles cannot come from the Cretaceous limestone forming the rockshelter, it seems clear that they were moved into the site from outside, either by natural processes or human action. According to Almeida et al. [65], these EE15 cobbles were collected by humans from the nearby formations and transported into the rockshelter for hearth construction due to their calorific value. As we have seen, the lithological composition of the cobble assemblage matches that of the surrounding alluvial deposits, especially those in Caranguejeira creek. This supports the strictly local origin of the cobbles and suggests that no significant lithological selection was made. However, the scarce presence in the archeological assemblage of the largest cobbles, which are well represented in the alluvial deposits, may suggest a certain size selection. This selection would be consistent with their transport to the shelter by humans, but we should also bear in mind the fact that most natural dynamics, including transport by water or gravity-driven processes, can also produce size sorting.

The transport into sites of huge quantities of cobbles, slabs and other types of stones for preparing occupation floors has been well documented in Upper Paleolithic sites [e.g., 78–81]. In general, these structures have been inferred from the appearance of discrete and continuous cobble layers. However, this is not the case at Lagar Velho, since the cobbles are widely scattered both horizontally and vertically. If the cobbles originally formed a single structure, this would indicate that they experienced significant post-depositional movement. Nevertheless, the refitting data suggests that, although movements took place in some instances, a generalized remobilization of the assemblage can be ruled out. In this context, the gradual introduction of the cobbles into the site during the formation of the assemblage seems more likely, a process that is at odds with the hypothesis of an anthropogenic structure. In terms of the association of cobbles with the layer affected by fire (i.e., layer 143), the unburnt elements predominate, even though cobbles were more likely to be exposed to fire than knapping products. However, we should bear in mind that, due to the lack of color changes and few macroscopically identifiable features, identifying burnt quartzite lithics with the naked eye remains difficult [82].

In this regard, the refitting of burnt fragments can be particularly informative. Although the temporal relationship between breakage and fire exposure cannot be determined in some cases, the most common pattern is that in which the cobbles were broken before they were exposed to fire and the fragments were differentially affected by thermal damage. This is particularly the case of the cobbles showing isolated removals. This is further evidence of the temporal depth of the archeological assemblage, since it marks the succession of the different events–firstly, there was cobble breakage or flake detachment, then fire exposure–throughout the formation process.

Another issue related to the cobble assemblage concerns the interpretation of these isolated removals produced by percussion. Different hypotheses can be envisaged to explain these lithics. Firstly, the detachments may have resulted from percussion activities. The accidental removal of flakes is one of the most diagnostic features observed in archeological hammerstones. However, these removals tend to be associated with other percussion traces, like cracks and pits [83,84]. The idea that flakes were accidentally detached while the cobbles were being used as hammerstones seems unlikely, as no other percussion marks are present. Secondly, these cobbles yielding only isolated removals could have resulted from tests made during raw material provisioning. The presence of tested cobbles is typical at sites that functioned as raw material provisioning locations. In these contexts, only a few removals are normally enough to check whether the blocks are suitable for knapping. Nevertheless, sites corresponding to lithic provisioning activities are normally associated with huge quantities of knapping products, including, for instance, cores in different stages of the reduction sequence. This is not the case of the layer 142/143 assemblage from Lagar Velho, where artifacts that can be clearly related to reduction sequences are relatively scarce. Intense knapping activities were recorded in layer EE15 at the “knapping hearth”, but this accumulation was stratigraphically above layers 142–143, where the bulk of lithic assemblage was found in the 2018–2021 campaign. Finally, the large accumulation of cobbles recorded in layers 142/143 may indicate a more complex mobility pattern, related to settlement dynamics and the technological organization of the hunter-gatherer groups. Cobbles could be transported and introduced into the site as raw material or as temporary deposits to be exploited later.

These above-mentioned flake detachment processes all resulted from unintentional or intentional human action. However, a third hypothesis must be addressed. These elements could also be geofacts derived from the natural fall of cobbles from the top of the cliff. According to Angelucci et al. [85], the formation of these layers coincides with intense erosion in the surroundings of the rockshelter. Almeida et al. [65] also indicated a linear concentration of cobbles in the northernmost part of EE15, located roughly below the main dripline, which would be consistent with the hypothesis that at least some cobbles fell from the top of the cliff where Cenozoic deposits overlie the Cretaceous limestones of the bedrock [69]. It has been pointed out [86–88]) that the impact of rocks falling from a height onto those located on the floor of caves and rockshelters may produce detachments that would exhibit attributes similar to those of intentional knapping. This process could produce blocks with single removals, but it would be also possible to produce blocks exhibiting several flake scars. In the case of Lagar Velho, these formation dynamics would be consistent with the higher densities of cobbles coinciding with the location of the dripline. Taking into account the equifinality of these processes, it seems hard to completely rule out the fact that some cobbles with isolated removals were the product of intentional percussion or raw material testing. Nevertheless, the contextual evidence suggests that a natural origin should be considered as the most parsimonious explanation for the majority of the removals observed on cobbles. Furthermore, it should be noted that the layer underlying 143 is a natural accumulation with no human activity. Its excavation is still in progress and the amount of sediment removed is low (less than in 143); however, cobbles similar to those found in 143 (including burning and flake removals) are also present in high numbers (NR = 56).

The products generated by these natural processes exhibit the characteristics defining intentional knapping (striking platform, well defined point and bulb of percussion, ventral and dorsal surfaces, etc.), making the distinction between artifacts and geofacts particularly difficult. This issue has been addressed by various authors [86,88–93]. These studies tend to indicate that understanding the formation processes specific to each site is essential for resolving this issue, since attribute analysis is not enough to confidently distinguish artifacts from geofacts, especially when flake detachments are related to natural percussion dynamics, as is the case in Lagar Velho. In this context, we must assume that some of the elements included in the ‘knapped assemblage’ were probably not produced by humans. In some cases, this has been confirmed by the refitting between flakes and cobbles with isolated removals. Other purported knapping products should perhaps be considered “incerto-facts” [94], a term used to designate those flakes whose agency–either natural or human–cannot be determined. We therefore think that a cautious approach to interpreting the Lagar Velho ‘knapped assemblage’ should be based on distinguishing two categories of lithics according to the reliability of their adscription to human agency.

Firstly, some lithics can be confidently assigned to intentional human knapping. Two main components can be distinguished in this assemblage. The first is made up of the artifacts included in the knapping refits and corresponding to the quartzite and quartz cobble reduction sequences. The series of contiguous and recurrent removals from the same striking platforms and the number of products are clear evidence of the intentional nature of these sequences. The second comprises the artifacts corresponding to exogeneous raw materials, absent from the geological formations contributing to the archeological deposit. This is principally the case of the twelve flint artifacts, but perhaps also the single rock crystal artifact (see Table 2). If we consider only these lithics, the anthropogenic assemblage is reduced to 45 artifacts.

While the first of these components is clearly associated with reduction sequences performed in the rockshelter, the origin of the second is more complex. The in-situ knapping of flint nodules was well attested to in EE15, at the “knapping hearth”, but it has not been documented in layer 142/143. Therefore, the origin of the flint artifacts from recent excavations remains elusive and various scenarios can be envisaged. At least two flint artifacts recovered from the periphery of the “knapping hearth” can be related to one of the reduction sequences carried out there. The other flint artifacts have not been associated with reduction sequences performed in the excavated area and therefore entered the assemblage as single items. However, it is difficult to determine whether they correspond either to knapping events carried out in areas not yet excavated or were transported into the rockshelter from outside.

For the remaining lithics showing attributes consistent with knapping, the human or natural agency should be considered in a probabilistic way. Those flakes presenting entirely cortical striking platforms and cortical dorsal faces are more likely to be the result of natural transformation processes. As the extension of dorsal cortex decreases, the number of dorsal scars increases and the platforms are no longer cortical, the human agency becomes more likely. However, it is important to remember that this reasoning does not imply that all the entirely cortical flakes are geofacts, since the use of cortical striking platforms also characterizes the knapping sequences carried out at Lagar Velho, meaning that some entirely cortical products were therefore detached at the beginning of those sequences. It should be highlighted that in this category of *more-or-less-likely* artifacts we must include one of the lithics classified as a core–the one that has not been refitted–and the two retouched flakes. The appearance of ‘pseudo-denticulates’ is one of the expected consequences of rockfall processes [88].

### Lithic technology

From a technological point of view, the lithic assemblage recovered from layers 142/143 exhibits the typical characteristics of expedient technologies, already recognized in EE15 [65]. The analysis of the cores and flakes, together with the information yielded by refits, indicates that the goal of the reduction sequences was the production of flakes using simple procedures. Knapping strategies were adapted to the morphology of raw material volumes and cortical surfaces were often used as striking platforms. Unidirectional detachments were the most common, although multidirectional strategies have also been documented. Although bipolar on anvil knapping has been sometimes identified in the reduction of quartzite and quartz cobbles [95,96], the typical features indicating the use of this technique (splintered pieces, bidirectional removals, crushing of the edges, square shape…) have not been identified in the Lagar Velho assemblage. The use of freehand knapping seems therefore the most likely scenario. No evidence suggesting core preparation or flake predetermination has been detected. Together with the predominant use of local raw materials, i.e., quartz and quartzite, this evidence is the perfect expression of a low-cost approach to lithic production, aimed at minimizing the time and effort invested in technology. In fact, the Lagar Velho assemblage can be considered one of the best examples of this kind of technology in Upper Paleolithic times.

These characteristics are consistent with those inferred from the study of the lithic assemblage recovered from the “knapping hearth” [65]. This assemblage also reflected the same low-cost approach to lithic production, including the predominance of unidirectional strategies and the use of cortical surfaces as striking platforms. However, we cannot overlook some interesting differences between the assemblage of the EE15 “knapping hearth” and that of layers 142/143. Firstly, flint artifacts formed a significant part of the EE15 assemblage. Although some flint artifacts were imported from outside, flint nodules were also reduced at the site. In contrast, flint represents a small portion of the lithic assemblage from the 142/143 excavation and no in situ debitage has been identified. It must be stressed that flint cannot be considered an exogenous raw material, since it is available a few kilometers from the site, even though flint nodules have never been documented from the alluvial deposits in Caranguejeira creek.

Secondly, some refitted sequences from the EE15 “knapping hearth” show extensive, intense exploitation with the production of tens of flakes. For instance, blocks QZI-1 and SVZ produced more than 200 artifacts [65]. Due to the large dimensions of these blocks, the products are variable in size, including large and very large flakes, even though small and very small products are always in the majority. Unlike these long productive sequences, the refits documented from layers 142/143 correspond to very short knapping events, in which a maximum of nine to ten products (refit 41) were detached. These differences are not related to the degree of exploitation, but rather to the size of the selected cobbles, which were fairly small in layers 142/143. For this reason, it seems clear that the events taking place around the EE15 “knapping hearth” and in layers 142/143 were similar in terms of reduction strategies and the expedient nature of the technical behavior, but quite different in terms of their techno-economic implications.

Layers 142/143 and EE15 yielded no diagnostic artifacts that permit us to assign a chrono-cultural context. The adscription of the Lagar Velho assemblages is restricted to the ^14^C dates available for these stratigraphic units, which indicate a Gravettian chronology (c. 29 ka cal. BP). Such an almost exclusive expedient character is uncommon in the panorama of the Gravettian industries from western Iberia, regardless of its chronology. The production of blades and especially bladelets is well documented throughout the Gravettian of Central Portugal, normally associated with the manufacture of backed implements [16,97,98]. In the Gravettian layers of Vale Boi, in southern Portugal, Marreiros et al. [20] highlighted the dominance of expedient reduction strategies based on the production of flakes from simple cores with one or two platforms. This behavior is even more remarkable in the Upper Paleolithic sites of the Côa valley, specifically those dated as Gravettian, where these expedient lithic industries involving local raw materials predominate in all the lithic assemblages known to date [e.g., 21]. Nevertheless, laminar production aimed at the manufacture of bladelets to be transformed into different types of points and barbs is always recorded in these cases. For this reason, it does not seem that an expedient stage can be defined in the Gravettian sequence of western Iberia, particularly in the Portuguese Estremadura where the microlithic component is systematically well represented. In this context, the appearance of an entirely expedient industry, like that of Lagar Velho, is very difficult to explain in chrono-cultural terms.

These peculiar characteristics of the Lagar Velho assemblage could be related to situational factors specific to the site and the functional nature of the human occupations. It seems clear that the expedient technical behavior cannot be explained by the adaptation to local raw materials. Firstly, the lithics recovered from the “knapping hearth” indicate that good-quality flint was worked with the same strategies used for quartz and quartzite. Secondly, there is evidence of the use of quartz in complex reduction sequences aimed at bladelet production in Gravettian assemblages from western Iberia, as can be seen, for instance, in the Terminal Gravettian of Lagar Velho layer TP06 [77]. To devise a functional explanation, related to the kind of activities carried out at the site, more information about other domains of the archeological record is needed. Almeida et al. [65] suggested that the faunal assemblage recovered from the EE15 surface inferred that skinning and hide processing were the main activities.

However, this hypothesis should be verified through a taphonomical and zooarcheological analysis of the bone remains from the ongoing excavations. Preliminary observations [99] from layer 143 have identified modifications produced by carnivores, digested and partially digested bones attributed to the bearded vulture [100] and a few striations that might correspond to anthropogenic marks. Fresh fractures in bones are the most abundant, but dry fractures due to fire action are also present. Thus, the zooarcheological results point to the involvement of complex taphonomic processes and biological agents in the accumulation and modification of bones. In any case, there is no evident relationship between these inferred activities and the use of expedient technologies, especially considering that the artifacts commonly used throughout the Upper Paleolithic for hide processing–endscrapers–are entirely absent from the assemblage.

To explain the singular characteristics of the Lagar Velho assemblage, we must address not only the presence of certain artifacts, but also the absence of others. The expediency was determined by the technical activities carried out at the site, but also by the absence of the more complex technical procedures typically documented in Gravettian industries. It can be hypothesized that the knapping events performed at the rockshelter are the partial expression of a technical system in which both expedient and more elaborate behaviors coexisted. A possible trace of this more elaborate component could be represented by the few laminar products identified in the assemblage (two bladelets in 142/143 and two blades in EE15), although these bladelets are not diagnostic enough to infer that real bladelet production methods were employed in another location by the humans who visited Lagar Velho.

When different degrees of technical complexity are present, we should consider the stochastic processes that conditioned the final composition of a lithic assemblage. In such a context, the probability that all levels of complexity are represented depends on the number of technical events–knapping sequences and transport of single items–making up the assemblage. At the same time, the number of technical events depends on both the intensity of the occupations and the temporal dimension of the archeological units. A complete picture of the technical systems would be more likely in a long-term occupation or well-developed palimpsest. From the technological standpoint, it seems that the human activities at Lagar Velho were sporadic, especially if we consider the lithics recovered in the 2018–2021 excavations. The EE15 excavation yielded many more artifacts, but most of them were clustered around the “knapping hearth”, suggesting that they were deposited in the same formation episode. The lithic record from Lagar Velho for the Late Gravettian seems, therefore, to correspond to high-resolution events, in the framework of an occupational pattern characterized by short and sporadic visits to the rockshelter, during which a limited number of production or import episodes took place. Given the rapid sedimentation rate in Lagar Velho rockshelter, the effects of large-superimposed events on the integrity and coherence of the archeological record are minor. The absence of some technical behaviors would be more likely in such a context, especially if those behaviors were not dominant in the technological repertoire of these human groups.

## Spatial and temporal patterns

This high-resolution pattern is still more evident if we try to locate the knapping events within the formation of the layers addressed in this paper. As we have seen, the refitting of burnt lithics offers a glimpse of the temporal dimension of the archeological assemblage. Considering exclusively the data from the lithics and taking the fire event/s as a reference, we can propose the following formation sequence, arranged from oldest to newest:

- The refits including both burnt and unburnt elements indicate that the processes that produced the breakage of these nodules occurred before the fire event/s. These refits correspond to broken cobbles and cobbles with isolated removals. This is the most common pattern, indicating that the majority of the cobbles accumulated in the first stages of the assemblage formation sequence. As we discussed earlier, cobbles falling from the top of the cliff is the most likely scenario to explain this breakage. Two flint artifacts also exhibit thermal damage, suggesting that at least some flint inputs took place before they were exposed to fire.
- For the refits where all the lithics show thermal damage, it is more difficult to establish the temporal relationship with the fire event/s. However, in most cases the lithics making up the refit exhibit the same degree of burning and some external surface alterations are continuous across the different fragments. This suggests that breakage took place after or during–maybe because of–burning.
- In sharp contrast with the broken cobbles and cobbles with removals, no burnt artifacts have been identified in the knapping refits. This indicates that most of the technical activities were performed after the fire event/s. Nevertheless, we cannot rule out the possibility that they were actually contemporaneous with the fire if these activities were carried out away from it or there was a reason for making sure the artifacts remained untouched by fire. However, the distribution of the refitted artifacts is no different from that of the burnt bones and cobbles. This pattern is like that observed around Almeida’s “knapping hearth” since the artifacts from the reduction sequences carried out there do not present thermal damage. In any case, this evidence suggests that the bulk of the human activities at the site took place during the late stages of the assemblage formation sequence.

The temporal relationship between the “knapping hearth” artifacts and the technical activities identified in layers 142/143 merits a final comment. From the stratigraphic perspective, the lithics from the “knapping hearth” are above the knapping sequences recorded in the new excavations. This indicates the persistence of expedient behaviors through time. These low-investment technologies were not employed in a single occupation episode but were maintained throughout a number of visits to the site. The conditions explaining this recurrence, whether functional or social, exerted their influence in different instances, strengthening the structural nature of these behaviors.

The refitting program developed at Lagar Velho fits with the field data and stratigraphic observations conducted during the fieldwork campaigns, suggesting that post-depositional disturbance was rare. The artifacts were clustered in archaeological layers and the refitting is in line with that previously described by Almeida et al. [65]. In summary, the internal coherence between the two layers and the contrasted relationship suggests that Layers EE15 (206 refits, 35% of the assemblage) and 142/143 (257 refits, 31.5%) correspond to two separate archaeological events. Only three refitted items link the two layers (EE15 and 143), which is statistically insignificant and could be related to excavation bias or natural sedimentological processes.

## Conclusions

The lithic assemblage from Layer EE15 of Lagar Velho rockshelter is one of the clearest examples of the use of expedient technologies from the Upper Paleolithic. In the context of the Gravettian chronology suggested by the radiometric dating, this entirely expedient character is particularly striking, since the Gravettian industries from Central Portugal are characterized by the presence of well-developed methods of blade and bladelet production. The analysis of the lithics recovered from layers 142/143 has confirmed the expedient nature of the technical behavior, although it has also indicated certain techno-economical differences. EE15 housed a huge accumulation of lithic remains–the “knapping hearth”–corresponding to different quartzite, flint, and quartz reduction sequences, some of them yielding hundreds of products. In contrast, only a few short quartzite and quartz reduction sequences have been identified in layers 142/143. These differences highlight the spatial variability in the distribution of human activities at Lagar Velho and show that the same expedient approach was used to achieve fairly different techno-economic goals. This study and refitting program carried out confirms the integrity of both archeological layers, i.e., EE15 and 142/143.

The European Upper Paleolithic sequence has been constructed over the last two centuries and is mostly based on the stratigraphic sequences identified in localities from the French Dordogne and neighboring areas. Lithic assemblages and the typological classification of stone-tools recovered from the most well-known sites allowed the classic sequence of Upper Paleolithic technocomplexes to be established, using the eponym French sites. The expedient nature of the lithics documented at the Lagar Velho rockshelter reveals that the Upper Paleolithic technocomplex scenario is much more diverse and heterogeneous than the classic sequence.

However, the study of the 142/143 assemblage addresses additional issues related to site formation processes and the distinction between geofacts and artifacts in certain archaeological contexts. Most of the assemblage is made up of cobbles of local raw materials, some of them broken or showing isolated removals. It was previously considered that these cobbles were brought into the rockshelter by humans and used in hearth construction. However, the absence of anthropic structures using cobbles and the features of the lithic assemblage observed in the present study point to a natural input as the most parsimonious explanation for the presence of these lithics. The 90 m drop from the top of the cliff to the site surface would have fractured some of the cobbles and produced detachment removals in others. This creates a challenging archeological problem as the flakes produced in these formation dynamics mimic the features derived from intentional human flaking. This problem is still more acute when we are dealing with expedient technologies, in which some procedures, like the common use of cortical surfaces as striking platforms, can make distinguishing geofacts from artifacts even more difficult. At Lagar Velho, the refitting of core reduction sequences has been essential for resolving this issue. However, some lithics are considered to be ‘incerto-facts’ due to the difficulty of establishing their human or natural agency.

Finally, by combining lithic refits and thermal damage, we have been able to shed some light on the temporal dimension of the assemblage and the role of the different agents throughout the formation sequence. While the natural inputs seem to be predominant in the first stages of the sequence, the human activities were more common in the later phases, after the fire event/s altered a large part of the assemblage. The combined use of these two temporal proxies has shown itself to be a powerful tool in palimpsest dissection. It is expected that the data from the lithics will provide insights into the interpretation of other archeological remains.

## References

[1] Andersson C, Read D. The Evolution of Cultural Complexity: Not by the Treadmill Alone. Curr Anthropol. 2016; 57(3): 261–286.

[2] Hoffecker JF, Hoffecker IT. The Structural and Functional Complexity of Hunter-Gatherer Technology. J Archaeol Method Theory. 2018; 25(1): 202–225.

[3] Muller A, Clarkson C, Shipton C. Measuring behavioural and cognitive complexity in lithic technology throughout human evolution. J Anthropol Archaeol. 2017; 48: 166–180.

[4] Vaesen K, Houkes W. Complexity and technological evolution: What everybody knows? Biol Philos. 2017; 32(6): 1245–1268. doi: 10.1007/s10539-017-9603-1 29563656 PMC5842246

[5] Alday A, editor. El Mesolítico de muescas y denticulados en la cuenca del Ebro y el litoral mediterráneo peninsular. Vitoria: Diputación Foral de Alava; 2006.

[6] Delagnes A, Rendu W. Shifts in Neandertal mobility, technology and subsistence strategies in western France. J Archaeol Sci. 2011; 38: 1771–1783.

[7] Holdaway S, Douglass M. A Twenty-First Century Archaeology of Stone Artifacts. J Archaeol Method Theory. 2012; 19: 101–131.

[8] McCall GS. Ethnoarchaeology and the Organization of Lithic Technology. J Archaeol Res. 2012; 20: 157–203.

[9] Vaquero M, Chacón MG, Cuartero F, García-Antón MD, Gómez de Soler B, Martínez K. The Lithic Assemblage of Level J. In: Carbonell E, editor. High Resolution Archaeology and Neanderthal Behaviour. Time and Space in Level J of Abric Romaní (Capellades, Spain). Dordrecht: Springer; 2012. pp. 189–311.

[10] Cazals N, Bartrolí R, Bon F, Bracco J-P, Clemente I, Fuertes Prieto N, et al. Des faciès et des hommes: réflexions sur les productions d’éclats au Paléolithique supérieur dans les Pyrénées françaises et espagnols. In: Territoires, déplacements, mobilité, échanges pendant la préhistoire: Terres et hommes du Sud: actes du 126e Congrès national des sociétés historiques et scientifiques, Toulouse, 2001. Paris: Ed. du CTHS; 2005. pp. 161–172.

[11] Chiotti L. La production d’eclats dans l’Aurignacien ancien de l’abri Pataud, les Eyzies-de-Tayac, Dordogne. Espacio, Tiempo y Forma. Serie I. Prehistoria y Arqueología. 2002; 15: 195–214.

[12] Clark GA, Barton CM. Lithics, landscapes & la Longue-durée—Curation & expediency as expressions of forager mobility. Quat Int. 2017; 450: 137–149.

[13] Cretin C, Le Licon-Julien G. Premières comparaisons sur la technologie du débitage du Magdalénien ancien: Les Jamblancs (Dordogne, France) et l’Abri Fritsch (Indre, France). Paleo. 1997; 9: 245–262.

[14] Pastoors A, Peresani M, editors. Flakes not Blades: The Role of Flake Production at the Onset of the Upper Palaeolithic in Europe. Mettmann: Wissenschaftliche Schriften des Neanderthal Museums; 2012.

[15] Zilhão J, Angelucci DE, Arnold LJ, d’Errico F, Dayet L, Demuro M, et al. Revisiting the Middle and Upper Palaeolithic archaeology of Gruta do Caldeirão (Tomar, Portugal). PLoS One. 2021; 16(10): e0259089. 34705887 10.1371/journal.pone.0259089 PMC8550450

[16] Zilhão J. O Paleolítico Superior da Estremadura Portuguesa. Lisboa: Edições Colibri; 1997.

[17] Gameiro C. La variabilité régionale des industries lithiques de la fin du Paléolithique supérieur au Portugal. PhD Thesis, Université Paris I, Panthéon-Sorbonne. 2012.

[18] Pereira T, Almeida F, Gibaja J, Holliday T, Bicho N. How they did it: Gravettian quartzite flakes from Western Iberia. In: Pastoors A, Peresani M, editors. Flakes not Blades: The Role of Flake Production at the Onset of the Upper Palaeolithic in Europe. Mettmann: Wissenschaftliche Schriften des Neanderthal Museums; 2012. pp. 25–50.

[19] Cascalheira J, Bicho N, Marreiros J, Pereira T, Évora M, Cortés M, et al. Vale Boi (Algarve, Portugal) and the Solutrean in Southwestern Iberia. Espacio, Tiempo y Forma. 2012; 5: 455–467.

[20] Marreiros J, Bicho N, Gibaja J, Pereira T, Cascalheira J. Lithic technology from the Gravettian of Vale Boi: new insights into Early Upper Paleolithic human behavior in Southern Iberian Peninsula. Quat Int. 2015; 359–360: 479–498.

[21] Aubry T. Os utensílios retocados e a economia da produção lítica. In: Aubry T, editor. 200 séculos da história do Vale do Côa: incursões da vida quotidiana dos caçadores-artistas do Paleolítico. Lisboa: IGESPAR; 2009. pp. 170–222.

[22] Aubry T, Barbosa AF, Luís L, Santos AT, Silvestre M. Quartz use in the absence of flint: Middle and Upper Palaeolithic raw material economy in the Côa Valley (North-eastern Portugal). Quat Int. 2016; 424: 113–129.

[23] Aubry T, Gameiro C, Mangado Llach J, Luís L, Matias H, do Pereiro T. Upper Palaeolithic lithic raw material sourcing in Central and Northern Portugal as an aid to reconstructing hunter-gatherer societies. J Lithic Stud. 2016; 3(2).

[24] Gaspar R, Ferreira J, Carrondo J, Silva MJ, García-Vadillo FJ. Open-air Gravettian lithic assemblages from Northeast Portugal: The Foz do Medal site (Sabor valley). Quat Int. 2016; 406: 44–64.

[25] Vaquero M, Alonso-Fernández ES. Technological changes and chrono-cultural boundaries: The role of expedient technologies in the upper paleolithic. J Archaeol Sci Rep. 2020; 31: 102346.

[26] Bosselin B, Djindjian F. Une révision de la séquence de la Riera (Asturies) et la question du Badegoulien cantabrique. Bull Soc Prehist Fr. 1999; 96(2): 153–173.

[27] Ducasse S, Langlais M. Entre Badegoulien et Magdalénien, nos cœurs balancent… Approche critique des industries lithiques du Sud de la France et du Nord-Est espagnol entre 19000 et 16500 BP. Bull Soc Prehist Fr. 2007; 104(4): 771–785.

[28] Fourloubey C. Badegoulien et premiers temps du Magdalénien. Un essai de clarification a l’aide d’un exemple, la vallée de l’Isle en Périgord. Paleo. 1998; 10: 185–209.

[29] Alday A. Los últimos cazadores-recolectores de la Iberia interior: La Alta-Media Cuenca del Ebro y la Meseta Norte. Munibe. 2002; 54: 79–101.

[30] Araújo AC, Almeida F, Valente MJ. Macrolithic industries of the Portuguese Mesolithic: a human adaptive response. In: McCartan S, Schulting R, Warren G, Woodman P, editors. Mesolithic Horizons. Papers presented at the Seventh International Conference on the Mesolithic in Europe, Belfast 2005. Oxford: Oxbow Books; 2009. pp. 779–787.

[31] Bicho NF. The End of the Paleolithic and the Mesolithic in Portugal. Curr Anthropol. 1994; 35(5): 664–674.

[32] Carvalho A. Novos dados sobre dois temas da Pré-História do Sul de Portugal: o Mirense e o processo de neolitização. Promontoria. 2007; 5: 91–110.

[33] Soto A, Alday A, Mangado X, Montes L. Epipaleolítico y Mesolítico en la vertiente sur de los Pirineos desde la perspectiva de la industria lítica. Munibe. 2016; 67: 295–312.

[34] Straus LG. Environmental and cultural changes across the Pleistocene-Holocene transition in Cantabrian Spain. Quat Int. 2018; 465: 222–233.

[35] Vaquero M, Esteban M, Allué E, Vallverdú J, Carbonell E, Bischoff JL. Middle Palaeolithic Refugium, or Archaeological Misconception? A New U-series and Radiocarbon Chronology of Abric Agut (Capellades, Spain). J Archaeol Sci. 2002; 29: 953–958.

[36] Straus LG, Clark GA, editors. La Riera Cave. Stone Age Hunter-Gatherer Adaptations in Northern Spain. Tempe: Arizona State University; 1986.

[37] Ríos J, de la Peña Alonso P, San Emeterio Gómez A. Estudio de las industrias líticas y óseas de la cueva de Aitzbitarte III (zona de la entrada). In: Altuna J, Mariezcurrena K, Ríos J, editors. Ocupaciones Humanas en Aitzbitarte III (País Vasco) 33.600–18.400 BP (Zona de entrada a la cueva). Vitoria: Gobierno Vasco; 2011. pp. 79–351.

[38] Martínez Fernández L. El Gravetiense en el sector occidental cantábrico y sus conexiones pirenaicas. Ph.D. Thesis, Universidad de Oviedo. 2015.

[39] Baena J, Carrión E. El nivel III de la cueva del Esquilleu (Castro-Cillórigo, Cantabria). Zephyrus. 2002; 55: 61–76.

[40] Baena J, Carrión E, Cuartero F, Fluck H. A chronicle of crisis: The Late Mousterian in north Iberia (Cueva del Esquilleu, Cantabria, Spain). Quat Int. 2012; 247: 199–211.

[41] Baena J, Carrión E, Torres C, Vaquero M. Mousterian inside the upper Paleolithic? The last interval of El Esquilleu (Cantabria, Spain) sequence. Quat Int. 2019; 508: 153–163.

[42] Coudart A. Entre Nouvelle-Guinée et Néolithique européen: de la correspondance entre les variations de l’architecture domestique, la durabilité culturelle et la cohésion sociale du groupe. In: Ethnoarchéologie: justification, problèmes, limites. Juan-les-Pins: Éditions APDCA; 1992. pp. 409–446.

[43] Keller CM, Keller JD. Cognition and tool use. The blacksmith at work. Cambridge: Cambridge University Press; 1996.

[44] Oswalt WH. Technological complexity: the Polar Eskimos and the Tareumiut. Arctic Anthropol. 1987; 24: 82–98.

[45] Schiffer MB. Studying Technological Change. A Behavioral Approach. Salt Lake City: The University of Utah Press; 2011.

[46] Bailey G. Time perspectives, palimpsests and the archaeology of time. J Anthropol Archaeol. 2007; 26: 198–223.

[47] Bailey G, Galadinou N. Caves, palimpsests and dwelling spaces: examples from the Upper Palaeolithic of south-east Europe. World Archaeol. 2009; 41(2): 215–241.

[48] Machado J, Hernández CM, Mallol C, Galván B. Lithic production, site formation and Middle Palaeolithic palimpsest analysis: in search of human occupation episodes at Abric del Pastor Stratigraphic Unit IV (Alicante, Spain). J Archaeol Sci. 2013; 40(5): 2254–2273.

[49] Malinsky-Buller A, Hovers E, Marder O. Making time: ‘Living floors’, ‘palimpsests’ and site formation processes–A perspective from the open-air Lower Paleolithic site of Revadim Quarry, Israel. J Anthropol Archaeol. 2011; 30(2): 89–101.

[50] Spagnolo V, Marciani G, Aureli D, Berna F, Boscato P, Ranaldo F, et al. Between hearths and volcanic ash: The SU 13 palimpsest of the Oscurusciuto rock shelter (Ginosa–Southern Italy): Analytical and interpretative questions. Quat Int. 2016; 417: 105–121.

[51] Vaquero M, Alonso S, García-Catalán S, García-Hernández A, Gómez de Soler B, Rettig D, et al. Temporal nature and recycling of Upper Paleolithic artifacts: the burned tools from the Molí del Salt site (Vimbodí i Poblet, northeastern Spain). J Archaeol Sci. 2012; 39(8): 2785–2796.

[52] Aubry T, Sampaio JD. Remontagem de rochas termoalteradas; um meio de reconstrução dos modos de funcionamento de estructuras de combustão no sítio de Olga grande 4 (Almendra, Vila Nova de Foz Côa). In: Mateus JE, Moreno-García M, editors. Paleoecologia Humana e Arqueociências, Um Programa Multidisciplinar para a Arqueologia soba Tutela da Cultura. Trabalhos de Arqueologia. 2003; 29: 331–335.

[53] Aubry T, Chauvière F-X, Sampaio JD. Informações obtidas com base nas remontagens da indústria de pedra lascada. In: Aubry T, editor. 200 séculos de história do Vale do Côa: Incursões na vida quotidiana dos caçadores artistas do Paleolítico. Lisboa: IGESPAR, IP; 2009. pp. 322–326.

[54] Araújo AC, Almeida F. L’apport de la méthode des remontages dans l’evaluation des processus de formation et d’altération des depots archéologiques: le cas de Barca do Xerez de Baixo (Portugal). In: Aubry T, Almeida F, Araújo AC, Tiffagom M, editors. International Union for Prehistoric and Protohistoric Sciences. Proceedings of the XV World Congress (Lisbon, 4–9 September 2006). Session C64. Space and Time: Which Diachronies, Which Synchronies, Which Scales? Session C65. Typology vs. Technology. Oxford: BAR International Series 183; 2008. pp. 91–99.

[55] Almeida F. 2008. Big puzzles, short stories: advantages of refitting for micro-scale spatial analysis of lithic scatters from Gravettian occupations in Portuguese Estremadura. In: Aubry T, Almeida F, Araújo AC, Tiffagom M, editors. International Union for Prehistoric and Protohistoric Sciences. Proceedings of the XV World Congress (Lisbon, 4–9 September 2006). Session C64. Space and Time: Which Diachronies, Which Synchronies, Which Scales? Session C65. Typology vs. Technology. Oxford: BAR International Series 183; 2008. pp. 69–79.

[56] Courbin P, Brenet M, Michel A, Gravina B. Spatial analysis of the late Middle Palaeolithic open-air site of Bout-des-Vergnes (Bergerac, Dordogne) based on lithic technology and refitting. J Archaeol Sci Rep. 2020; 32: 102373.

[57] Hallos J. “15 Minutes of Fame”: Exploring the temporal dimension of Middle Pleistocene lithic technology. J Hum Evol. 2005; 49(2): 155–179. doi: 10.1016/j.jhevol.2005.03.002 15964609

[58] Karlin C, Julien M. An autumn at Pincevent (Seine-et-Marne, France): refitting for an ethnographic approach of a Magdalenian settlement. Archaeol Anthropol Sci. 2019; 11(9): 4437–4465.

[59] Machado J, Mayor A, Hernández CM, Galván B. Lithic refitting and the analysis of Middle Palaeolithic settlement dynamics: a high-temporal resolution example from El Pastor rock shelter (Eastern Iberia). Archaeol Anthropol Sci. 2019; 11(9): 4539–4554.

[60] Mugaj J. 2021. Seasonal Aggregation Site in Late Paleolithic—Intrasite Analysis of Large Hamburgian Encampment in Myszęcin, Western Poland. Lithic Technol. 2021; 47(2): 106–116.

[61] O’Brien M. Evaluating the Contemporaneity of Households at the Eden-Farson site. Int J Osteoarchaeol. 2015; 25(5): 653–664.

[62] Olive M, Pigeot N, Bignon-Lau O. Un campement magdalénien à Étiolles (Essonne). Des activités à la microsociologie d’un hábitat. Gallia Prehist. 2019; 59: 47–108.

[63] Vaquero M, Romagnoli F, Bargalló A, Chacón MG, Gómez de Soler B, Picin A, et al. Lithic refitting and intrasite artifact transport: a view from the Middle Paleolithic. Archaeol Anthropol Sci. 2019; 11(9): 4491–4513.

[64] Bordes F. Question de contemporanéité. L’illusion des remontages. Bull Soc Prehist Fr. 1980; 77(5): 132–133.

[65] Almeida F, Moreno-García M, Angelucci DE. From under the bulldozer’s claws: the EE15 Late Gravettian occupation surface of the Lagar Velho rockshelter. World Archaeol. 2009; 41(2): 242–261.

[66] Zilhão J, Trinkaus E, editors. Portrait of the Artist as a Child. The Gravettian Human Skeleton from the Abrigo do Lagar Velho and its Archeological Context. Trabalhos de Arqueologia 22. Lisboa: Instituto Português de Arqueologia; 2002.

[67] Duarte C, Maurício J, Pettitt PB, Souto P, Trinkaus E, van der Plicht H, Zilhão J. The early Upper Paleolithic human skeleton from the Abrigo do Lagar Velho (Portugal) and modern human emergence in Iberia. Proc Natl Acad Sci USA. 1999; 96(13): 7604–7609 doi: 10.1073/pnas.96.13.7604 10377462 PMC22133

[68] Araújo AC, Costa AM. Relatório dos trabalhos realizados no Abrigo do Lagar Velho, Vale do Lapedo. Trabalhos do LARC, 3. Lisboa: DGPC; 2013.

[69] Angelucci D. The Geoarcheological Context. In: Zilhão J, Trinkaus E, editors. Portrait of the Artist as a Child. The Gravettian Human Skeleton from the Abrigo do Lagar Velho and its Archeological Context. Trabalhos de Arqueologia 22. Lisboa: Instituto Português de Arqueologia; 2002. pp. 58–91.

[70] Araújo AC, Costa AM, Sanz M, Lucena A, Daura J. O Abrigo do Lagar Velho Revisitado. In: Arnaud J, Neves C, Martins A, editors. Arqueologia em Portugal 2023 –Estado da Questão. Lisboa: Associação dos Arqueólogos Portugueses; 2023. pp. 61–74.

[71] Bronk Ramsey C. Bayesian analysis of radiocarbon dates. Radiocarbon. 2009; 51: 337–360.

[72] Reimer PJ, Austin WEN, Bard E, Bayliss A, Blackwell PG, Bronk Ramsey C, et al. The IntCal20 northern hemisphere radiocarbon age calibration curve (0–55 cal kBP). Radiocarbon. 2020; 62: 725–757.

[73] Fripp JB, Diplas P. Surface sampling in gravel streams. J Hydraul Eng. 1993; 119: 473–490.

[74] Laplace G. La typologie analytique et structurale: Base rationnelle d’étude des industries lithiques et osseuses. Banques de données archéologiques. Colloques nationaux du CNRS. 1972; 932: 91–143.

[75] Cziesla E. On refitting of stone artifacts. In: Cziesla E, Eickhoff S, Arts N, Winter D, editors. The Big Puzzle. International Symposium on Refitting Stone Artefacts. Monrepos 1987. Studies in Modern Archaeology 1. Bonn: Holos; 1990. pp. 9–44.

[76] Aubry T, Mangado Llach J, Matias H. Matérias-primas das ferramentas em pedra lascada da Pré-história do Centro e Nordeste de Portugal. In: Dinis PA, Gomes A, Monteiro Rodrigues S, editors. Proveniência de materiais geológicos: abordagens sobre o Quaternário de Portugal. Coimbra: Associação Portuguesa para o Estudo do Quaternário; 2014. pp. 165–192.

[77] Almeida F, Gameiro C, Zilhão J. The Artifact Assemblages. In: Zilhão J, Trinkaus E, editors. Portrait of the Artist as a Child. The Gravettian Human Skeleton from the Abrigo do Lagar Velho and its Archeological Context. Trabalhos de Arqueologia 22. Lisboa: Instituto Português de Arqueologia; 2002. pp. 202–219.

[78] Aubry T, Sampaio JD, Chauvière F-X. As outras categorias de vestígios líticos. In: Aubry T, editor. 200 séculos da história do Vale do Côa: incursões da vida quotidiana dos caçadores-artistas do Paleolítico. Lisboa: IGESPAR; 2009. pp. 269–326.

[79] Aubry T, Luís L, Mangado Llach J, Matias H. Adaptation to Resources and Environments during the Last Glacial Maximum by Hunter Gatherer Societies in Atlantic Europe. J Anthropol Res. 2015; 71: 521–544.

[80] Petraglia MD, Akoshima K, Straus LG. Interpreting the Formation of the Abri Dufaure: An Upper Paleolithic Site in Southwestern France. J Anthropol Archaeol. 1994; 13: 139–151.

[81] Yar B, Dubois Ph. Les structures d’habitat au Paléolithique en France. Montagnac: Éditions Monique Mergoil; 1999.

[82] Sanz M, Daura J, Cabanes D, Égüez N, Carrancho A, Badal E, et al. Early evidence of fire in south-western Europe: the Acheulean site of Gruta da Aroeira (Torres Novas, Portugal). Sci Rep. 2020; 10(1): 12053. doi: 10.1038/s41598-020-68839-w 32694542 PMC7374095

[83] Adams JL. Ground stone tools. A technological approach. Salt Lake City: University of Utah Press; 2002.

[84] de Beaune SA. Les Galets Utilisés au Paléolithique Supérieur. Paris: CNRS; 1997.

[85] Angelucci DE, Almeida F, Zilhão J. La sucesión estratigráfica del abrigo de Lagar Velho (valle de Lapedo, Portugal): significado paleoambiental y ocupación antrópica. In: Santonja M, Pérez-González A, Machado MJ, editors. Geoarqueología y patrimonio en la Península Ibérica y el entorno mediterráneo. Soria: ADEMA; 2005. pp. 359–368.

[86] Borrazzo K. Estimating the Contribution of Lithic Pseudo Artifacts to the Archaeological Record: Actualistic Taphonomic Research at Casa de Piedra de Roselló 1 (Chubut, Argentina). Ethnoarchaeol. 2022; 14(2): 136–159.

[87] Meltzer DJ, Adovasio JM, Dillehay TD. On a Pleistocene human occupation at Pedra Furada, Brazil. Antiquity. 1994; 68: 695–714.

[88] Nash DT. Distinguishing Stone Artifacts from Naturefacts created by Rockfall Processes. In: Goldberg P, Nash DT, Petraglia MD, editors. Formation Processes in Archaeological Context. Madison: Prehistory Press; 1993. pp. 125–138.

[89] Bradbury AP. Modern or prehistoric: experiments in distinguishing between culturally and mechanically produced chipped stone artifacts. North Am Archaeol. 2001; 22: 231–258.

[90] Gillespie JD, Tupakka S, Cluney C. Distinguishing Between Naturally and Culturally Flaked Cobbles: A Test Case from Alberta, Canada. Geoarchaeol. 2004; 19(7): 615–633.

[91] Lubinski PM, Terry K, McCutcheon PT. Comparative methods for distinguishing flakes from geofacts: a case study from the Wenas Creek Mammoth site. J Archaeol Sci. 2014; 52: 308–320.

[92] Peacock E. Distinguishing between Artefacts and Geofacts: A Test Case from Eastern England. J Field Archaeol. 1991; 18(3): 345–361.

[93] Schnurrenberger D, Bryan AL. A Contribution to the Naturefact/Artifact Controversy. In: Plew MG, Woods JC, Pavesic MG, editors. Stone Tool Analysis. Albuquerque: University of New Mexico Press; 1985. pp. 133–159.

[94] Roebroeks W, Stapert D. On the “Lower Palaeolithic” site La Belle Roche: an alternative interpretation. Curr Anthropol. 1986; 27: 369–371.

[95] De la Peña P. A qualitative guide to recognize bipolar knapping for flint and quartz. Lithic Technol. 2015; 40: 316–331.

[96] Roda Gilabert X, Mora R, Martínez-Moreno J. Identifying bipolar knapping in the Mesolithic site of Font del Ros (northeast Iberia). Philos. Trans. R. Soc. B. 2015; 370: 20140354. doi: 10.1098/rstb.2014.0354 26483532 PMC4614717

[97] Aubry T, Zilhão J, Almeida F. À propos de la variabilité technique et culturelle de l’entité gravettienne au Portugal: bilan des dernières découvertes et perspectives de recherche. Paleo. 2007; 19: 53–72.

[98] Bicho N, Haws J, Marreiros J. Desde el Mondego al Guadiana: la ocupación gravetiense de la fachada atlántica portuguesa. In: de las Heras C, Lasheras JA, Arrizabalaga A, de la Rasilla M, editors. Pensando el Gravetiense: nuevos datos para la región cantábrica en su contexto peninsular y pirenaico. Santander: Museo y Centro de Investigación de Altamira; 2012. pp. 55–72.

[99] Vettese D, Sanz M, Daura J, Costa AM, Araújo AC. Spatial analyses of the distribution of taphonomic marks on long animal bones of Lagar Velho Rock-shelter (Early Gravettian), in Lapedo Valley (Leiria, Portugal). J Taphonomy. 2022; 16: 205–206.

[100] Sanz M, Daura J, Costa AM, Araújo AC. The characterization of bearded vulture (Gypaetus barbatus) coprolites in the archaeological record. Sci Rep. 2023; 13: 57. doi: 10.1038/s41598-022-25288-x 36596809 PMC9810590

